# Optimal Time of Collapse to Return of Spontaneous Circulation to Apply Targeted Temperature Management for Cardiac Arrest: A Bayesian Network Meta-Analysis

**DOI:** 10.3389/fcvm.2021.784917

**Published:** 2022-01-07

**Authors:** Jingwei Duan, Qiangrong Zhai, Yuanchao Shi, Hongxia Ge, Kang Zheng, Lanfang Du, Baomin Duan, Jie Yu, Qingbian Ma

**Affiliations:** ^1^Emergency Department, Peking University Third Hospital, Beijing, China; ^2^First Clinical Medicine School, Lanzhou University, Lanzhou, China; ^3^Emergency Department, Kaifeng Center Hospital, Kaifeng, China; ^4^The George Institute for Global Health, The University of New South Wales Sydney, Sydney, NSW, Australia; ^5^Faculty of Medicine, University of New South Wales, Sydney, NSW, Australia; ^6^Department of Cardiology, Peking University Third Hospital, Beijing, China

**Keywords:** cardiac arrest, targeted temperature management, return of spontaneous circulation (ROSC), survival, good neurological outcome

## Abstract

**Background:** Both the American Heart Association (AHA) and European Resuscitation Council (ERC) have strongly recommended targeted temperature management (TTM) for patients who remain in coma after return of spontaneous circulation (ROSC). However, the role of TTM, especially hypothermia, in cardiac arrest patients after TTM2 trials has become much uncertain.

**Methods:** We searched four online databases (PubMed, Embase, CENTRAL, and Web of Science) and conducted a Bayesian network meta-analysis. Based on the time of collapse to ROSC and whether the patient received TTM or not, we divided this analysis into eight groups (<20 min + TTM, <20 min, 20–39 min + TTM, 20–39 min, 40–59 min + TTM, 40–59 min, ≥60 min + TTM and ≥60 min) to compare their 30-day and at-discharge survival and neurologic outcomes.

**Results:** From an initial search of 3,023 articles, a total of 9,005 patients from 42 trials were eligible and were included in this network meta-analysis. Compared with other groups, patients in the <20 min + TTM group were more likely to have better survival and good neurologic outcomes (probability = 46.1 and 52.5%, respectively). In comparing the same time groups with and without TTM, only the survival and neurologic outcome of the 20–39 min + TTM group was significantly better than that of the 20–39 min group [odds ratio = 1.41, 95% confidence interval (1.04–1.91); OR = 1.46, 95% CI (1.07–2.00) respectively]. Applying TTM with <20 min or more than 40 min of collapse to ROSC did not improve survival or neurologic outcome [ <20 min vs. <20 min + TTM: OR = 1.02, 95% CI (0.61–1.71)/OR = 1.03, 95% CI (0.61–1.75); 40–59 min vs. 40–59 min + TTM: OR = 1.50, 95% CI (0.97–2.32)/OR = 1.40, 95% CI (0.81–2.44); ≧60 min vs. ≧60 min + TTM: OR = 2.09, 95% CI (0.70–6.24)/OR = 4.14, 95% CI (0.91–18.74), respectively]. Both survival and good neurologic outcome were closely related to the time from collapse to ROSC.

**Conclusion:** Survival and good neurologic outcome are closely associated with the time of collapse to ROSC. These findings supported that 20–40 min of collapse to ROSC should be a more suitable indication for TTM for cardiac arrest patients. Moreover, the future trials should pay more attention to these patients who suffer from moderate injury.

**Systematic Review Registration:** [https://inplasy.com/?s=202180027], identifier [INPLASY202180027]

## Introduction

Cardiac arrest (CA) has a variety of causes and severely threatens human life. Even when cardiopulmonary resuscitation (CPR) is performed promptly and with high quality under the latest guidelines, only ~10.4% of patients survive after out-of-hospital cardiac arrest (OHCA), and 25.8% survive after in-hospital cardiac arrest (IHCA) (1). Even with such a low survival rate, only 8–21% of patients have good neurologic outcome at discharge (2). Therefore, improving survival and neurologic outcome has remained a challenge in clinical practice.

The term *hypothermia* can be traced back to an ancient “neoteric” technology first used by ancient Egyptians, Greeks, and Romans to induce cooling for battle-inflicted trauma and a variety of cerebral disturbances (3). In recent decades, this technology has been applied to improve survival and neurologic outcome and has given rise to a new concept, targeted temperature management (TTM), for patients with CA (4). However, there is a debate about TTM that has not stopped. Two early randomized controlled trials (RCTs), as well as a more recent trial, have consistently shown that TTM could improve clinical outcomes for CA (5–7). Additionally, TTM has been recommended by both the American Heart Association (AHA) and European Resuscitation Council (ERC) in their latest guidelines for adults who do not follow physicians' orders after return of spontaneous circulation (ROSC) from OHCA or IHCA with any initial rhythm and control the temperature within 32–36°C (2, 8). TTM might improve ischemia-perfusion by reducing cell metabolism and thus improve clinical outcomes (9). While clinicians firmly believe that targeted hypothermia is beneficial for eligible patients, a recent blockbuster study has disrupted that belief. A recent RCT showed that targeted hypothermia did not improve clinical outcomes compared with targeted normothermia, whether survival or neurologic (10). Therefore, should we apply TTM? Or does it matter when we apply TTM? The results of the latest trial are difficult to deny. Thus, we conducted the current network meta-analysis to identify the suitable time of collapse to ROSC, which is an optimal indication for TTM to improve survival and neurologic outcome.

## Methods

### Protocol Registration

We registered the protocol for this systematic review with the International Platform of Registered Systematic Review and Meta-analysis Protocols (registration number: INPLASY202180027).

### Databases and Search Strategy

We performed this network meta-analysis by searching four online databases (PubMed, Embase, CENTRAL, and Web of Science). References of relevant meta-analyses, letters, editorials, reviews, and eligible trials were also screened. The initial search was broad, and no limitations were made regarding publication type, study data type, language, or species. Our detailed search strategy is shown in the Supplementary Material.

Initially, two trained investigators screened the titles and abstracts of all articles independently. If they were of different opinions, a third person intervened to settle the disagreements. Second, after preliminary screening, the remaining studies were further screened by reading the full text. Finally, the eligibility of all included trials was confirmed by contacting the corresponding authors.

### Criteria for Inclusion and Exclusion

After the screening, trials that met the following criteria were included: (a) patients in the trial underwent CA, whether IHCA or OHCA; (b) valid 30-day or at-discharge survival neurologic outcome data could be extracted; (c) the time of collapse to ROSC could be extracted so we could divide patients into groups based on that data; (d) patients who received TTM should have a temperature below 36°C.

If a trial met any one of the following criteria, it was excluded from our analysis: (a) only special populations were included in the study, such as pediatric or obstetric; (b) full text was not available; (c) the article was written in a language other than English; (d) the trial performed on animals or cells; (e) collapse with no witness and without reliable approach to recode the time of collapse to ROSC. We did not limit inclusion to randomized controlled trials; if a retrospective or cohort trial met the inclusion criteria, it was included in our analysis. While meta-analyses and reviews were not included, we reviewed their references and included trials that met the inclusion criteria.

### Data Extraction and Quality Assessment

Published data from the included trials were pooled in this network meta-analysis, and a standard method was used to extract patients' demographics and trial characteristics. Finally, two pre-specified endpoints, 30-day or at-discharge survival and neurologic outcome, were collected from the included trials.

Cochrane Handbook version 5.1.0 was used to assess the quality of each RCT and its risk of bias (Supplementary Figures 9, 10). The Newcastle–Ottawa Assessment Scale (NOS) for control and cohort studies was used to assess the quality and risk of bias of each retrospective and cohort study, respectively (Supplementary Tables 5, 6). We considered that a NOS score greater than seven was high quality (11).

### Endpoints and Assignment Definitions

We distributed the studies in this analysis according to two endpoints: 30-day or at-discharge survival and good neurologic outcome. Based on the Cerebral Performance Category (CPC) scale (which ranges from 1 to 5, with higher scores indicating greater disability), a good neurologic outcome was defined as CPC 1–2 (12, 13). The center temperature controlled below 36°C was considered to be receiving TTM.

Based on the time of collapse to ROSC and whether a patient received TTM, we assigned patients to eight groups (<20 min + TTM, <20 min, 20–39 min + TTM, 20–39 min, 40–59 min + TTM, 40–59 min, ≥60 min + TTM and ≥60 min) to compare them and determine an optimal indication for TTM.

### Synthesis and Analysis

We conducted this analysis based on the Preferred Reporting Items for Systematic Reviews and Meta-Analyses (PRISMA) guidelines. A Bayesian random effects survival model for analyzing multiple treatment groups was built to compare the different groups regarding their 30-day or at-discharge survival endpoints and neurologic outcome endpoints. We used a Bayesian extension of the hierarchical random effects model proposed by Lumley for networks of multiarm trials. A Bayesian network meta-analysis can make direct or indirect comparisons to determine whether there is a direct comparison between two groups. Pooled data were analyzed *via* STATA/MP 16.0 (Stata Corp LP, College Station, Texas, USA). Markov chain Monte Carlo samplers were run in Stata, and 4 chains were run with different starting values. Vague, non-informative prior distributions with very small precision were given. A burn-in phase of 20,000 iterations was used to ensure convergence. The convergence was checked by running 4 chains at different starting values using the Gelman-Rubin methods, which were stable in all instances. For inference, 50,000 iterations were used. A fixed effect model was used in traditional frequentist meta-analysis to analyze pooled odds ratios (ORs), so a traditional frequentist meta-analysis was performed in this study using STATA. Pairwise ORs were estimated from the median of the posterior distribution with credible intervals (CIs) taken from the 2.5 and 97.5% percentiles. The results are considered significantly different when CI did not include 1 and the OR > 1 means favor last or OR <1 means favor first. Markov chain Monte Carlo (MCMC) modeling was used to calculate the relative ranking probability of each intervention. “Rankograms” along with the surface under the cumulative ranking curve (SUCRA) were employed to compare the hierarchy of efficacy and safety of the interventions (14). SUCRA is a numeric representation of the overall ranking and assigns a single number associated with each treatment. SUCRA values range from 0 to 100%. The higher the SUCRA value, and the closer to 100%, the higher the likelihood that a group is in the top rank, or highly effective; the closer the SUCRA value is to 0, the more likely the group is in the bottom rank, or ineffective. Additionally, a funnel plot was constructed to assess the trials' publication bias (Supplementary Figures 4, 8). If the funnel plot was not evenly distributed, we considered publication bias to be evident in those trials. Nodesplitting models were constructed to assess the level of inconsistency between the direct and indirect evidence estimates (Supplementary Figures 2, 6). Global inconsistency was used to assess global inconsistency. When *P* > 0.05, we considered that there was no inconsistency (Supplementary Figures 1, 5). Moreover, the loop inconsistency test was used to assess the inconsistency of every closed loop. When the 95% confidence interval included 1, we considered that there was no inconsistency in this closed loop (Supplementary Tables 2, 4). A netweight plot was used to assess the weight of pairwise direct and indirect comparisons between different interventions (Supplementary Figures 3, 7).

## Results

**Figure 1:**
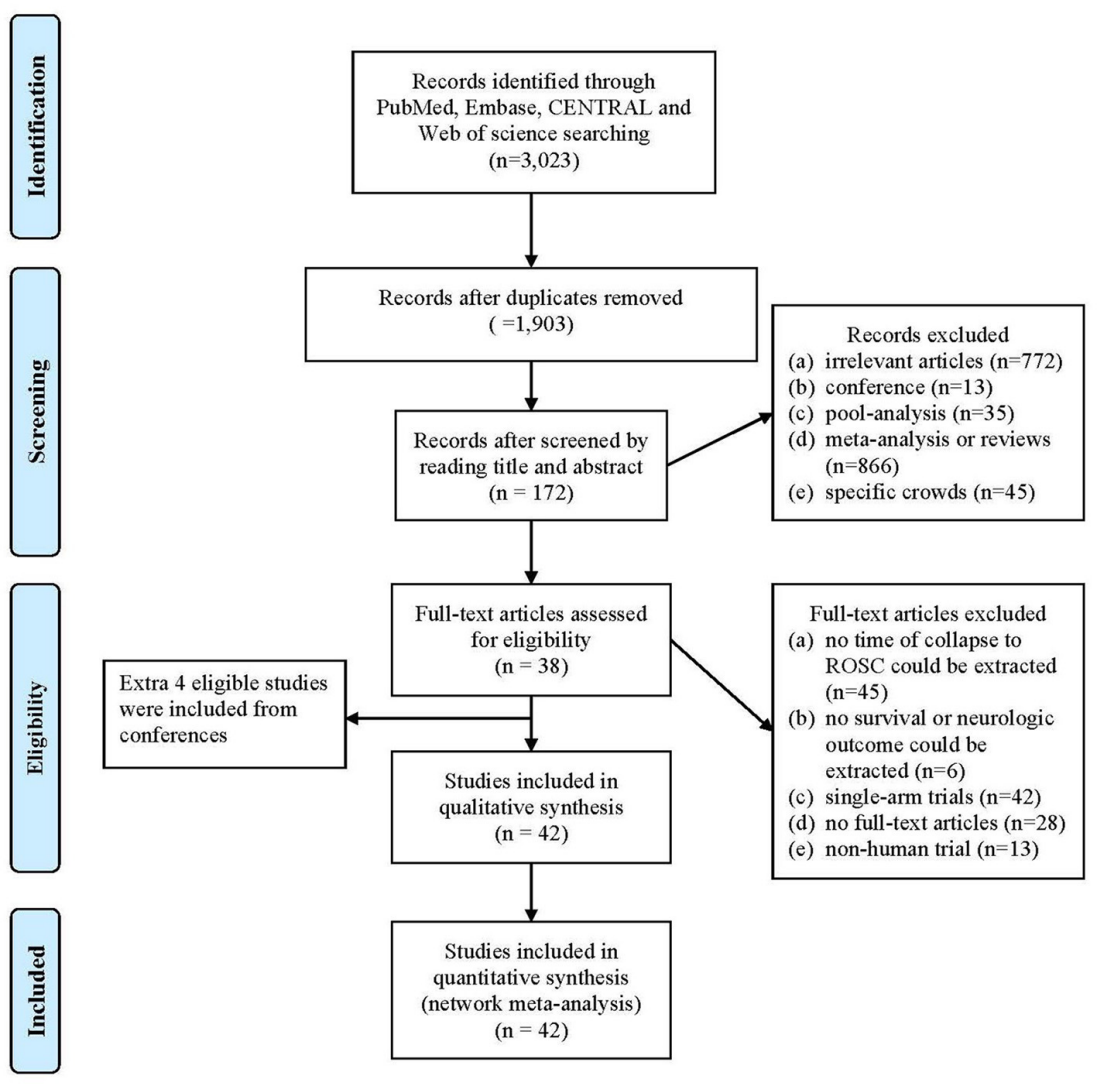
Flow diagram according to PRISMA statement.

Following our search strategy, an initial 3,023 articles were identified from four online databases. A total of 1,120 duplicate articles were removed. The remaining 1,903 articles were screened by reading the titles and abstracts, after which 937 additional articles were removed because they did not meet the inclusion and exclusion criteria. The full texts of 172 articles were acquired and further screened based on the inclusion and exclusion criteria. Finally, with the addition of 4 studies acquired by reviewing the articles' references, 42 studies were included in this analysis (Figure 1).

### Characteristics of Included Trials

A total of 9,005 patients from 42 trials were included in this network meta-analysis to compare their 30-day or at-discharge survival and their neurologic outcome. Only nine of the total included trials were RCTs, with the others being retrospective or cohort trials. Of the 9,005 patients, 5,622 (62%) received TTM, of which the main cooling method was core cooling. The temperature of patients who received TTM ranged between 32 and 36°C. The vast majority of all patients were male (7,103/9,005, 79%). Survival data were available for 8,878 of the 9,005 patients, and neurologic outcome was available for 8,123 patients. The survival rate of all patients was 37% (3,315/8,878), and the rate of good neurologic outcome was 33% (2,712/8,123). Other detailed characteristics of the included trials are shown in Tables 1, 2.

**Table 1:** Characteristics of included trials (RCT: randomized controlled trial).

**Author name**	**Year**	**Site**	**Design of trial**	**Follow-up (day)**	**Survival outcome**	**Neurologic outcome**
Agarwal et al.	2018	US	Retrospective	365	Yes	Yes
Arrich et al.	2007	Europe	Retrospective	At discharge	Yes	Yes
Blumenstein et al.	2015	Germany	Retrospective	365	Yes	Yes
Castren et al.	2010	Sweden	RCT	At discharge	Yes	Yes
Chen et al.	2008	China	Prospective	365	Yes	No
Choi et al.	2016	Korea	Cohort	30	Yes	Yes
Chou et al.	2012	China	Prospective	365	Yes	No
De Fazio et al.	2019	Belgium	Retrospective	180	Yes	Yes
Dankiewicz et al.	2021	Multicenter	RCT	180	Yes	Yes
Ferreira et al.	2009	Netherland	Retrospective	At discharge	Yes	Yes
Fink et al.	2008	Germany	Retrospective	30	Yes	No
Fjølner et al.	2016	Denmark	Retrospective	At discharge	Yes	No
Gillies et al.	2010	UK	Retrospective	At discharge	No	Yes
Goto et al.	2018	Japan	Retrospective	30	Yes	Yes
Hachimi-Idrissi et al.	2005	Belgium	RCT	30	Yes	Yes
Han et al.	2015	Korea	Retrospective	30	Yes	Yes
Holzer et al.	2002	Multicenter	RCT	180	Yes	Yes
Jouffroy et al.	2017	France	Prospective	28	Yes	Yes
Kagawa et al.	2010	Japan	Retrospective	365	Yes	Yes
Kagawa et al.	2012	Japan	Retrospective	30	Yes	Yes
Kagawa et al.	2015	Japan	Prospective	90	Yes	Yes
Kamarainen et al.	2009	Finland	RCT	At discharge	Yes	Yes
Kim et al.	2007	US	Prospective	At discharge	Yes	No
Kim et al.	2014	Korea	Retrospective	180	Yes	Yes
Kim et al.	2018	Korea	Retrospective	At discharge	Yes	Yes
Look et al.	2017	Singapore	RCT	30 or discharge	Yes	Yes
Maekawa et al.	2013	Japan	Prospective	180	Yes	Yes
Mecklenburg et al.	2020	Germany	Retrospective	28	Yes	No
Nagao et al.	2010	Japan	Retrospective	365	Yes	Yes
Nielsen et al.	2013	Multicenter	RCT	180	Yes	Yes
Okada et al.	2011	Japan	Retrospective	At discharge	No	Yes
Otani et al.	2018	Japan	Retrospective	At discharge	Yes	Yes
Pang et al.	2016	Singapore	RCT	180	Yes	Yes
Pang et al.	2017	Singapore	Retrospective	At discharge	Yes	Yes
Ryu et al.	2019	Korea	Retrospective	30	Yes	Yes
Scales et al.	2017	Canada	RCT	At discharge	Yes	Yes
Schenfeld et al.	2015	US	Retrospective	365	Yes	Yes
Schober et al.	2017	Austria	Cohort	At discharge	Yes	Yes
Shin et al.	2013	Korea	Cohort	730	Yes	No
Sonder et al.	2018	Multicenter	Prospective	At discharge	Yes	Yes
Tømte et al.	2011	Norway	Cohort	365	Yes	Yes
Yukawa et al.	2017	Japan	Retrospective	At discharge	No	Yes

**Table 2:** Characteristics of comparison arms (A: <20 min + TTM; B: <20 min; C: 20–39 min + TTM; D: 20–39 min + TTM; E: 20–39 min; F: 40–59 min + TTM; G: ≥60 min + TTM; H: ≥60 min.

**Author name**	**Time of collapse to ROSC of comparison arm (min)**	**Comparison arm**	**TTM case**	**Method of cooling**	**Temperature control (°C)**	**Male** ***n* (%)**	**Mean age (years)**	**OHCA** ***n* (%)**	**Witnessed arrest *n* (%)**	**Shockable rhythm** ***n* (%)**	**Bystander CPR *n* (%)**	**ACS cause CA** ***n* (%)**
Agarwal et al.	15 vs. 20	A vs. C	385 (100)	Core	33–36	206 (54)	65	280 (73)	100 (26)	80 (21)	88 (23)	72 (19)
Arrich et al.	27 vs. 23	C vs. D	462 (79)	Core or surface	32–34	433 (74)	60	484 (83)	531 (90)	366 (62)	283 (48)	446 (76)
Blumenstein et al.	33 vs. 40	D vs. F	N/A	N/A	N/A	195 (60)	75	0	324 (100)	9 (3)	324 (100)	225 (69)
Castren et al.	18 vs. 30	A vs. C	93 (48)	Core	34	146 (75)	65	194 (100)	194 (100)	59 (30)	79 (41)	164 (85)
Chen et al.	53 vs. 43	E vs. F	59 (34)	Core	34	123 (72)	59	0	172 (100)	55 (32)	172 (100)	117 (68)
Choi et al.	16 vs. 13 vs. 22 vs. 20	A vs. B vs. C vs. D	16 (27)	Core	33	45 (75)	59	60 (100)	60 (100)	16 (27)	49 (82)	N/A
Chou et al.	15 vs. 30 vs. 42 vs. 66	B vs. D vs. F vs. H	N/A	N/A	N/A	57 (86)	64	0	66 (100)	35 (53)	66 (100)	66 (100)
De Fazio et al.	19 vs. 20	A vs. C	352 (100)	Core or surface	32–34	293 (83)	62	352 (100)	323 (92)	312 (89)	293 (83)	191 (54)
Dankiewicz et al.	35 vs. 35	C vs. D	1,861 (100)	Core	33	1,477 (79)	63	1,861 (100)	1,702 (91)	1,371 (74)	1,487 (80)	N/A
Ferreira et al.	8 vs. 10	A vs. B	49 (65)	Core or surface	32	25 (33)	64	75 (100)	N/A	25 (33)	55 (73)	25 (33)
Fink et al.	18 vs. 22	A vs. C	59 (100)	Surface	33	29 (59)	63	49 (100)	42 (86)	35 (71)	40 (82)	40 (82)
Fjølner et al.	54 vs. 70	F vs. H	N/A	N/A	N/A	12 (57)	48	21 (100)	21 (100)	14 (67)	21 (100)	12 (57)
Gillies et al.	19 vs. 22	A vs. C	34 (100)	Core or surface	32–36	63 (76)	61	73 (88)	83 (100)	53 (64)	83 (100)	N/A
Goto et al.	57 vs. 59	E vs. F	63 (44)	Core	34	122 (85)	63	144 (100)	25 (18)	88 (61)	54 (38)	100 (69)
Hachimi-Idrissi et al.	35 vs. 34	C vs. D	16 (48)	Surface	33	21 (64)	73	33 (100)	18 (55)	28 (85)	5 (15)	N/A
Han et al.	76 vs. 64	G vs. H	26 (26)	Core	32–34	74 (74)	55	75 (75)	86 (86)	54 (54)	73 (73)	N/A
Holzer et al.	21 vs. 22	C vs. D	137 (50)	Surface	32–34	210 (76)	59	275 (100)	273 (99)	275 (100)	127 (46)	51 (19)
Jouffroy et al.	36 vs. 40	C vs. E	39 (100)	Core	32–34	30 (65)	52	46 (100)	N/A	N/A	N/A	27 (59)
Kagawa et al. (2010)	17 vs. 22 vs. 43 vs. 40	B vs. C vs. E vs. F	25 (32)	Core	33–34	55 (71)	62	39 (51)	67 (87)	29 (38)	63 (82)	43 (56)
Kagawa et al. (2012)	45 vs. 55	E vs. F	32 (37)	Core	34	70 (81)	63	42 (49)	77 (90)	46 (53)	67 (80)	86 (100)
Kagawa et al. (2015)	32 vs. 43	C vs. E	237 (100)	Core	32–36	180 (76)	61	193 (81)	193 (81)	126 (53)	127 (54)	76 (32)
Kamarainen et al.	22 vs. 23	C vs. D	19 (51)	Core	33–36	35 (95)	61	37 (100)	29 (78)	28 (76)	15 (41)	32 (86)
Kim et al. (2007)	47 vs. 51	E vs. F	63 (50)	Core	33–36	88 (70)	66	125 (100)	88 (70)	51 (41)	54 (43)	N/A
Kim et al. (2014)	37 vs. 21	C vs. D	88 (18)	Core	33	326 (65)	67	499 (100)	371 (74)	116 (23)	174 (35)	226 (45)
Kim et al. (2018)	47 vs. 44	E vs. F	25 (25)	Surface	33–34	69 (68)	55	22 (22)	101 (100)	45 (45)	98 (97)	N/A
Look et al.	26 vs. 24	C vs. D	45 (52)	Core	34	69 (79)	64	72 (83)	65 (75)	9 (10)	25 (29)	21 (24)
Maekawa et al.	49 vs. 56	E vs. F	33 (20)	Core	34	123 (76)	64	162 (100)	162 (100)	56 (35)	71 (44)	N/A
Mecklenburg et al.	16 vs. 12	A vs. B	36 (55)	Core	32–34	46 (70)	51	N/A	N/A	N/A	N/A	27 (41)
Nagao et al.	58 vs. 64	E vs. G	177 (100)	Core	34	148 (84)	59	177 (100)	94 (53)	143 (81)	94 (53)	131 (74)
Nielsen et al.	25 vs. 25	C vs. D	473 (50)	Core	33 or 36	761 (81)	64	939 (100)	838 (89)	729 (78)	683 (73)	N/A
Okada et al.	17 vs. 35	A vs. C	40 (100)	Surface	34.5–35.5	53 (80)	59	66 (100)	57 (86)	52 (79)	27 (41)	44 (68)
Otani et al.	23 vs. 25 vs. 40 vs. 44	C vs. D vs. E vs. F	28 (21)	Core	34	115 (85)	65	135 (100)	135 (100)	87 (64)	74 (55)	64 (47)
Pang et al. (2016)	30 vs. 22	C vs. D	9 (43)	Core	34	17 (81)	53	2 (8)	19 (91)	7 (33)	21 (100)	N/A
Pang et al. (2017)	31 vs. 35	C vs. D	14 (18)	Core	34	62 (79)	50	6 (7)	73 (92)	33 (42)	79 (100)	62 (79)
Ryu et al.	19 vs. 38	B vs. D	N/A	N/A	N/A	174 (64)	63	24 (8)	272 (99)	79 (29)	266 (97)	104 (38)
Scales et al.	16 vs. 16	A vs. B	279 (48)	Core	33–36	380 (65)	68	582 (100)	351 (60)	258 (44)	270 (46)	N/A
Schenfeld et al.	17 vs. 20	A vs. C	132 (100)	Core	33	82 (62)	58	132 (100)	111 (84)	83 (63)	94 (75)	11 (8)
Schober et al.	17 vs. 17 vs. 55 vs. 55	A vs. B vs. E vs. F	51 (21)	Core	34–36	178 (74)	60	239 (100)	210 (88)	138 (58)	73 (31)	N/A
Shin et al.	32 vs. 31	C vs. D	85 (21)	Core	34	254 (63)	61	406 (100)	406 (100)	98 (24)	406 (100)	120 (30)
Sonder et al.	18 vs. 23	A vs. C	7 (6)	Core or surface	33–34	55 (46)	60	89 (74)	100 (83)	54 (45)	65 (54)	37 (31)
Tømte et al.	25 vs. 28	C vs. D	73 (45)	Core or surface	34	137 (85)	58	162 (100)	141 (87)	120 (74)	125 (77)	131 (71)
Yukawa et al.	33 vs. 45	D vs. F	N/A	N/A	N/A	65 (82)	59	79 (100)	21 (27)	58 (73)	46 (58)	39 (49)

*CA, cardiac arrest; TTM, targeted temperature management; core cooling involved endovascular cooling, intravenous cold fluids, automated peritoneal lavage, any dialysis technique, extracorporeal membrane oxygenation, esophageal or transnasal; surface cooling involved skin exposure, cooling beds, iced packs, cooling pads, air-circulating or water-circulating blankets, water-filled blankets, air-filled blankets*.

### Survival Outcome

**Figure 2:**
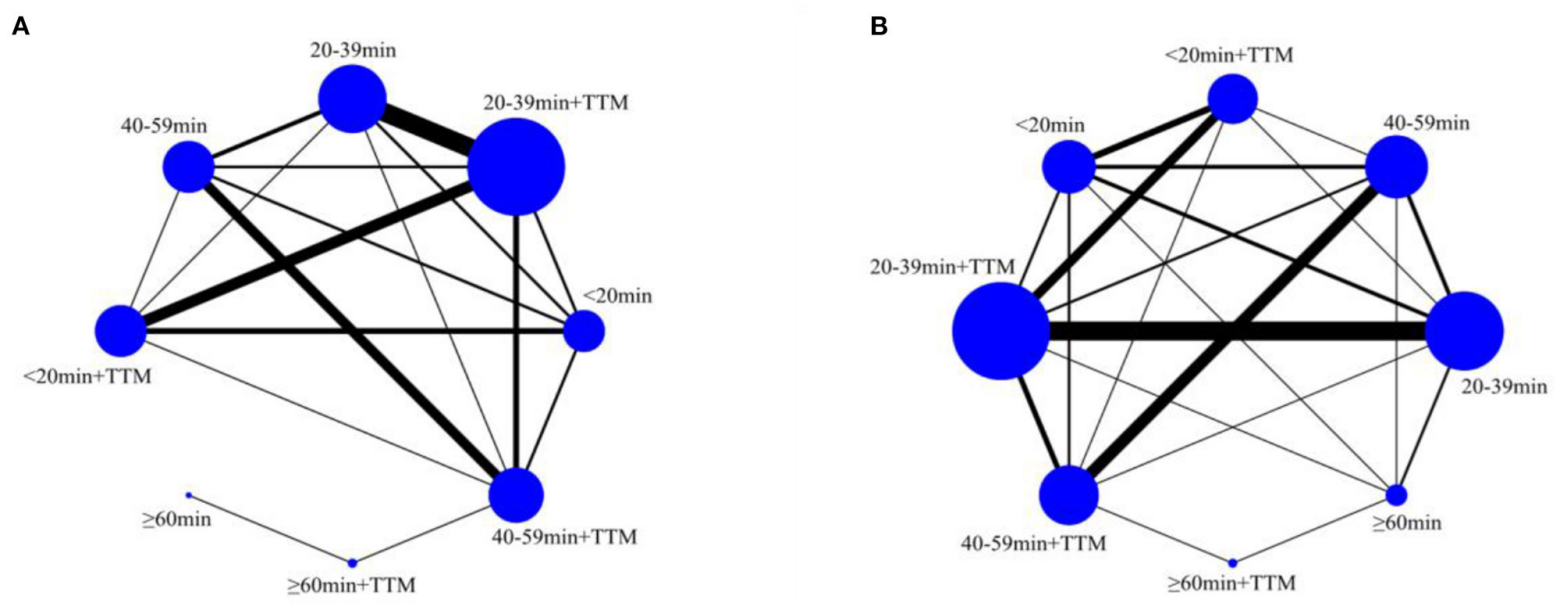
Network plot for 30-day or at-discharge survival **(A)** and good neurologic outcome **(B)**.

A total of 8,778 patients from 39 trials were included to compare survival (958 patients in the <20 min + TTM group from 11 trials; 713 patients in <20 min from 8 trials; 2,792 patients in 20–39 min + TTM from 23 trials; 2,941 patients in 20–39 min from 17 trials; 464 patients in 40–59 min + TTM from 12 trials, 609 patients in 40–59 min from 12 trials, 185 patients in ≧60 min + TTM from 2 trials and 116 patients in ≧60 min from 3 trials (Figure 2A).

**Figure 3:**
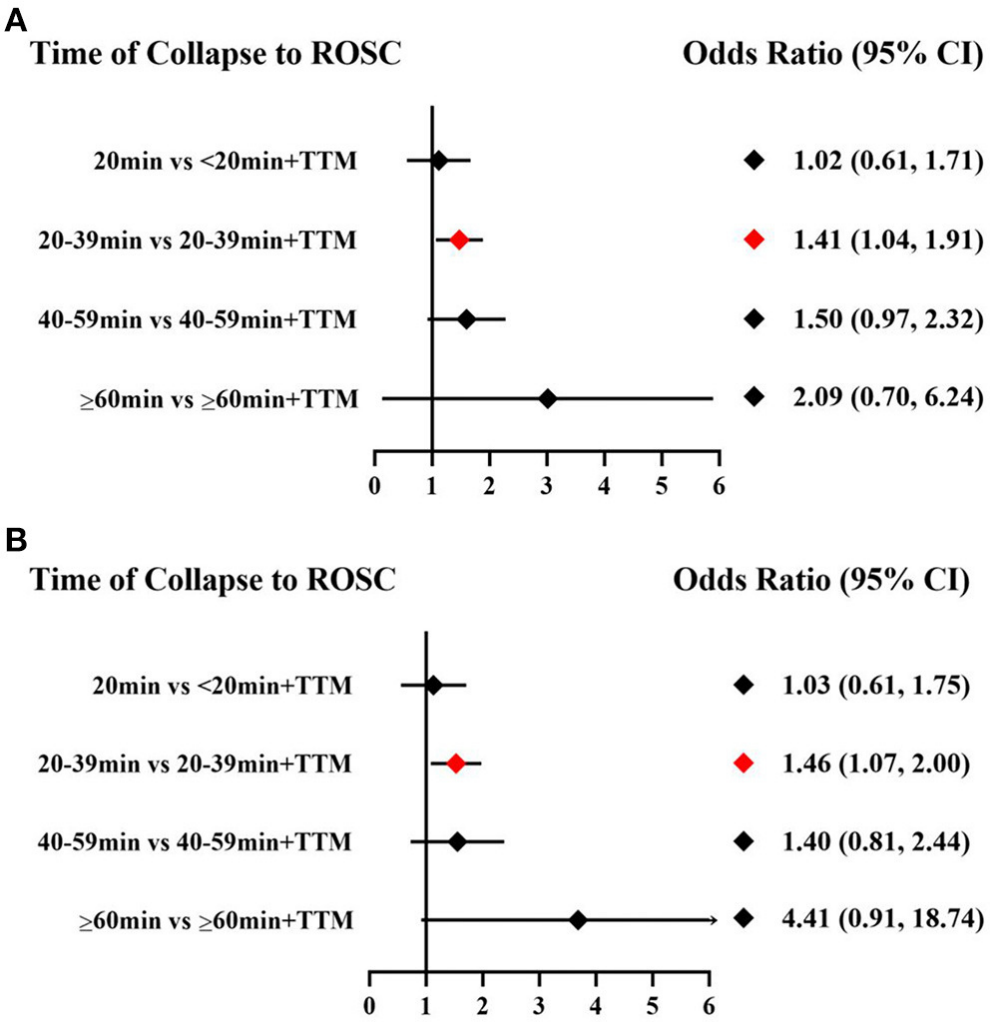
Forest plot for survival **(A)** and good neurologic outcome **(B)** with the same time of collapse to ROSC.

**Figure 4:**
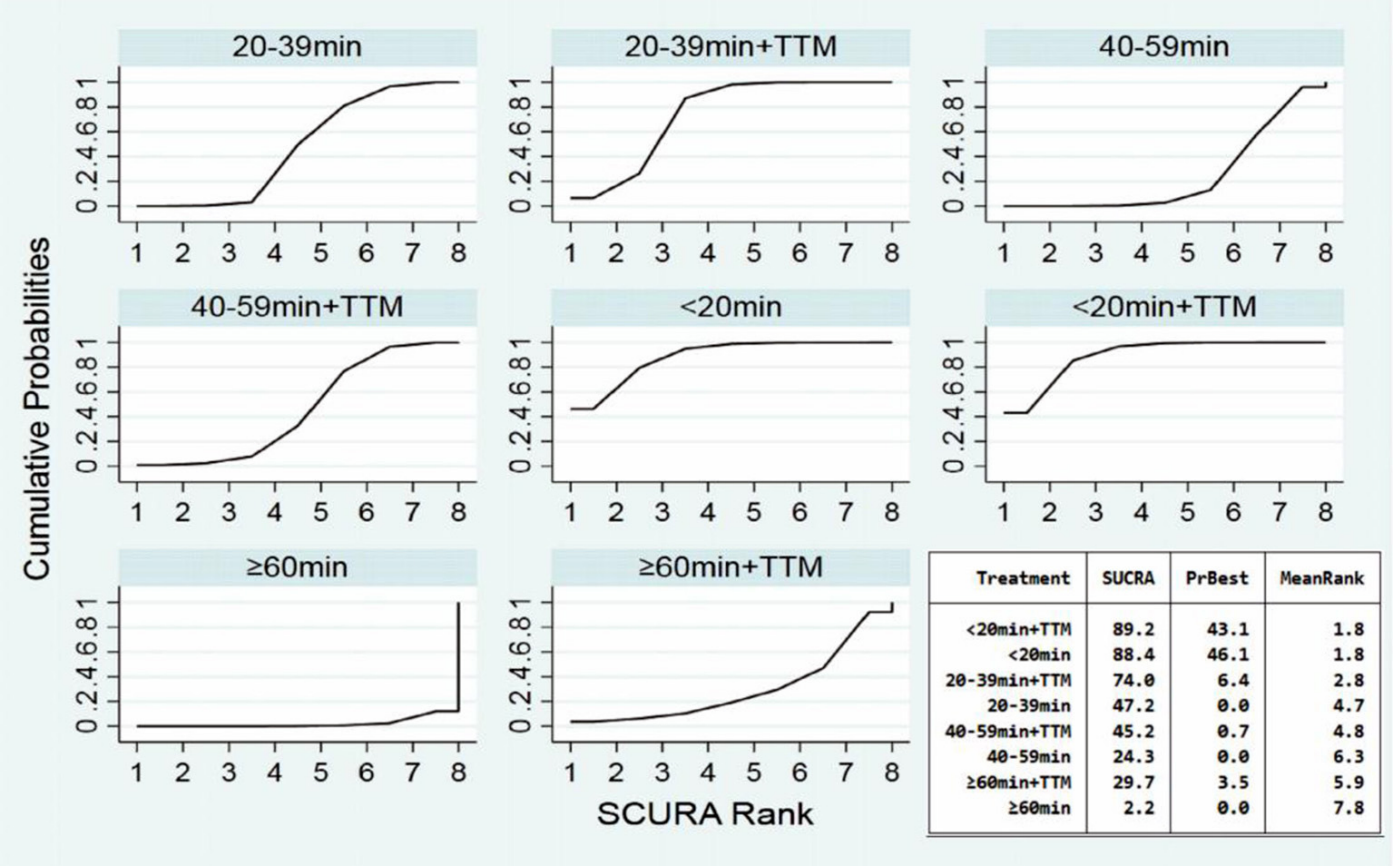
SUCRA plot for survival (the area of SUCRA is shown in the lower right corner).

**Figure 5:**
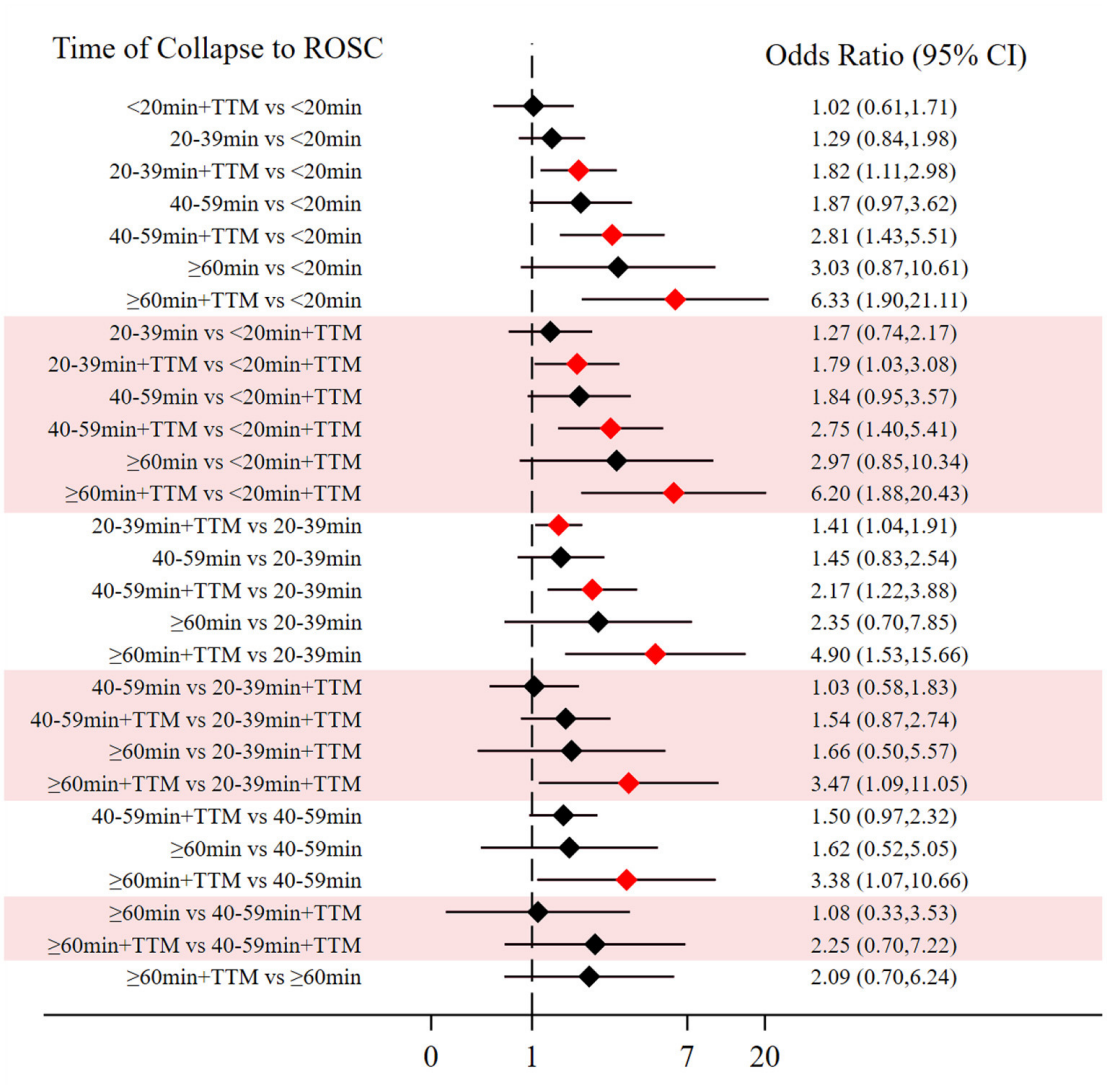
Forest plot for survival (the stepped distributions of each group's comparison with the same given group are shown in the same background color).

Comparing the influence of the application of TTM in patients with the same time of collapse to ROSC, the 20–39 min + TTM group showed a significant difference from the 20–39 min [OR = 1.41, 95% CI (1.04–1.91)] group, but TTM resulted in no significant difference among the other groups (Figure 3A). Based on a forest plot of survival, we found a stepped comparative distribution among the different groups (which are the same background color in **Figure 5** forest plot), with the survival of patients related to the time of collapse to ROSC. Based on the rank and cumulative probability, patients in the <20 min + TTM group had the best probability of survival outcome (probability = 46.1%, SUCRA = 89.2) (Figure 4; Supplementary Table 1). We also found that there were significant differences among the other non-TTM groups in their comparisons with <20 min + TTM [20–39 min: OR = 1.82, 95% CI (1.11–2.98); 40–59 min: OR = 2.81, 95% CI (1.43–5.51); ≥60 min: OR = 6.33, 95% CI (1.90–21.11)]. When the time of collapse to ROSC exceeds 40 min, applying TTM might not improve survival, as only ≥60 min + TTM vs. 40–59 min showed a difference [OR = 3.38, 95% CI (1.07–10.66)] (Figure 5).

### Good Neurologic Outcome

A total of 8,123 patients from 35 trials were included to compare neurologic outcome: 969 patients in the <20 min + TTM group from 11 trials; 671 patients in <20 min from 6 trials; 2,744 patients in 20–39 min + TTM from 23 trials; 2,589 patients in 20–39 min from 16 trials; 342 patients in 40–59 min + TTM from 10 trials, 549 patients in 40–59 min from 9 trials, 185 patients in ≧60 min + TTM from 2 trials and 74 patients in ≧60 min from 1 trial (Figure 2B).

**Figure 6:**
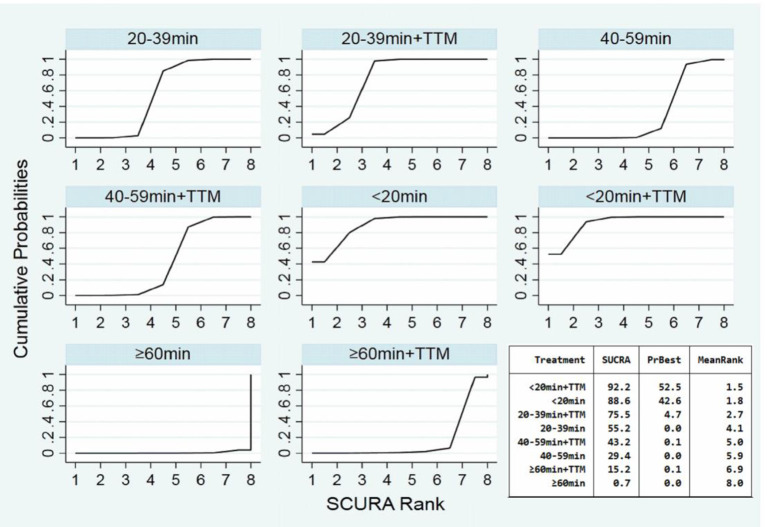
SUCRA plot for good neurologic outcome (the area of SUCRA is shown in the lower right corner).

**Figure 7:**
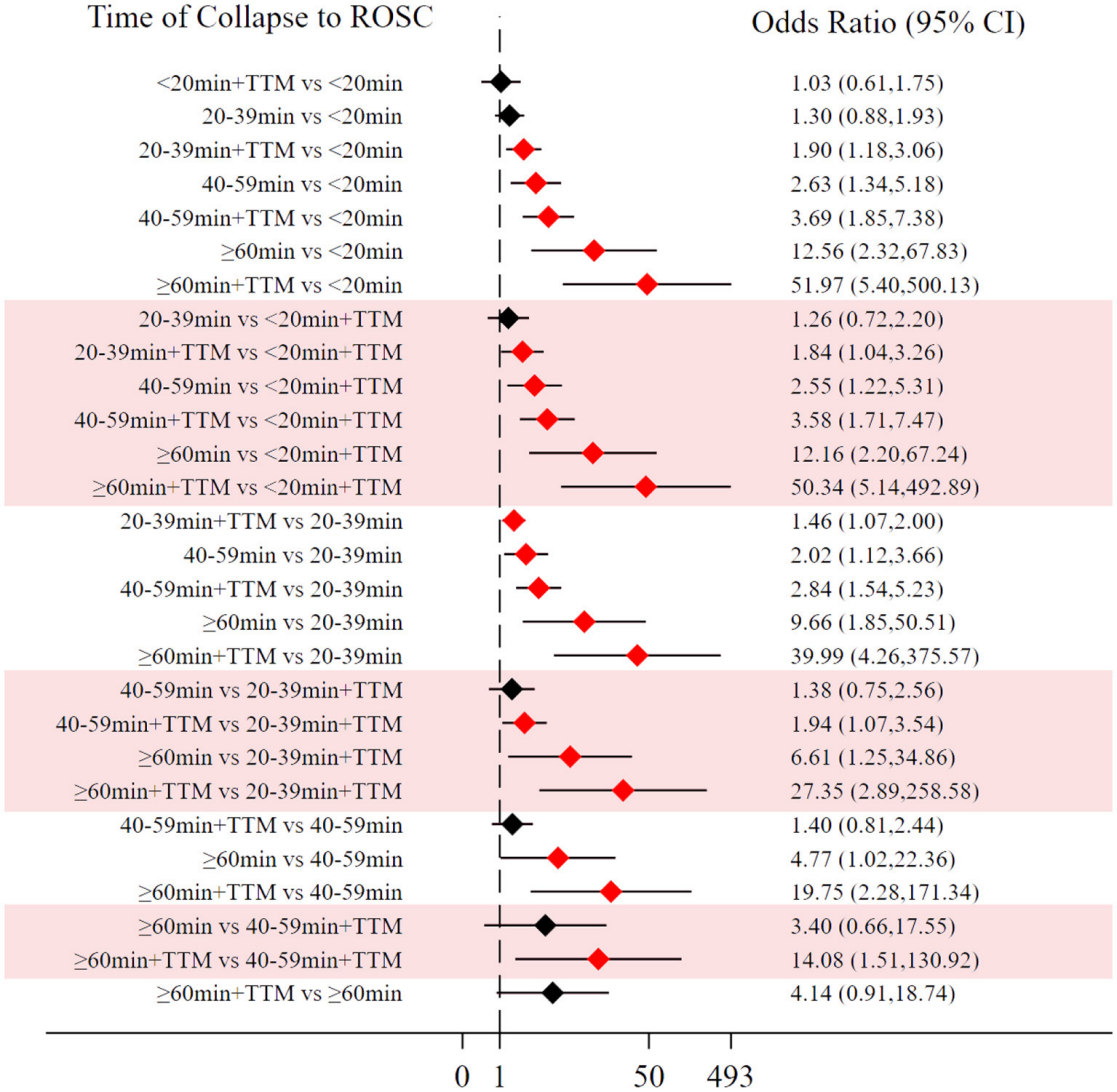
Forest plot for good neurologic outcome (the stepped distributions of each group's comparison with the same given group are shown in the same background color).

Comparing the influence of the application of TTM in patients with the same time of collapse to ROSC, the 20–39 min + TTM group showed a significant difference from the 20–39 min [OR = 1.46, 95% CI (1.07–2.00)] group, but TTM resulted in no significant difference among the other groups (Figure 3B). Based on a forest plot of neurologic outcome, we found a stepped comparative distribution among the different groups (the same background color in **Figure 7** forest plot), with the neurologic outcome of patients related to the time of collapse to ROSC. Based on the rank and cumulative probability, patients in the <20 min + TTM group had the best probability of good neurologic outcome (probability = 52.5%, SUCRA = 92.2) (Figure 6; Supplementary Table 3). There were significant differences between the <20 min + TTM group and the other groups [20–39 min: OR = 1.90, 95% CI (1.18–3.06); 40–59 min + TTM: OR = 3.69, 95% CI (1.85–7.38); 40–59 min: OR = 2.63, 95% CI (1.34–5.18); ≥60 min + TTM: OR = 51.97, 95% CI (5.40–500.13); ≥60 min: OR = 12.56, 95% CI (2.32–67.83)] (Figure 7).

### Sensitive Analysis

To detect the potential bias in this network meta-analysis, we conducted extra sensitivity analysis (15). First, we noticed that three trials included only IHCA (16–18). Thus, we consider that patients in these trials might receive CPR of higher quality by medical care personnel than OHCA by bystanders. In addition to the three trials, one trial with no record of OHCA and two trials with <10% OHCA were excluded from the sensitivity analysis (19–21). From this sensitivity analysis, we reached similar results of survival and good neurologic outcome: patients in 20–39+min TTM group had better clinical outcomes than patients in the 20–39 min without TTM group [survival: OR = 1.48, 95% CI (1.06–2.07); good neurologic outcome: OR = 1.38, 95% CI (1.01–1.88)] (Supplementary Figures 11, 12). Acute coronary syndrome (ACS) is one of most common causes of CA, and a previous trial showed that it might have better clinical outcomes than other diseases causing CA (22). To eliminate the potential bias that this factor may cause, we excluded 6 trials that included only ACS patients or did not record the cause of CA, which were not declared in the trial or protocol (18, 20, 23–26). From this sensitivity analysis, we reached similar results of survival and good neurologic outcome: patients in the 20–39 min TTM group had better clinical outcomes than patients in the 20–39 min without TTM group [survival: OR = 1.46, 95% CI (1.05–2.05); good neurologic outcome: OR = 1.35, 95% CI (1.01–1.80)] (Supplementary Figures 13, 14).

## Discussion

From this network meta-analysis, we found that both survival and good neurologic outcome were related to the time of collapse to ROSC. However, as this has long been clinicians' consensus, the most significant finding is that TTM did improve short-term survival and neurologic outcome for patients with CA and that improvement is also related to the time of collapse to ROSC. The positive effect of TTM takes place between 20 and 40 min of collapse to ROSC. A shorter or longer interval of collapse to ROSC applying the procedure does not appear to significantly improve survival or neurologic outcome, especially within 20 min of collapse to ROSC. Additionally, from the forest plots, we noticed that the stepped distribution was more apparent in improving neurologic outcome than survival. Therefore, we speculated that the main effect of TTM might be to improve short-term neurologic outcome for patients with CA.

Although both the AHA and ERC have recommended TTM for patients who are still in comas after ROSC from OHCA or IHCA with any initial rhythm, in clinical practice (2, 8), clinicians still hesitate to apply TTM; in particular, the latest RCT did not support this recommendation (10). What, then, is causing this hesitation? There are two main ways to implement TTM: core and surface (27). Regardless of the cooling method, the ultimate goal is to keep the core temperature at a certain level (28). A recent systematic review showed that, compared with surface cooling, core cooling could improve neurologic outcome for patients with CA (29). However, based on evidence in the currently reviewed studies, core cooling methods do not improve either survival or neurologic outcome (28, 30) and might incur more frequent bleeding complications for patients with CA (31), as confirmed by a recent meta-analysis (32). Therefore, the cooling methods do not seem to affect the outcomes of TTM for CA.

Another question related to clinician hesitation: does a difference in temperature level have any effect on the CA patient's clinical outcome? The recent RCT noted above has given us a definite answer. The researchers compared the mortality and neurologic outcome in CA patients at 33 and 36°C and found no significant difference at 180 days [mortality: hazard ratio = 1.06, 95% CI (0.89–1.28), *p* = 0.51; and poor neurologic outcome: risk ratio = 1.02, 95% CI (0.88–1.16), *p* = 0.78] (33). In addition to the two factors just discussed, temperature level and cooling method, there are other technological or methodological factors that might have influenced the clinical outcomes of applying TTM in the RCT, such as pre- or post-hospital cooling, local cooling, duration of TTM, and rate and extent of cooling and reheating. With regard to these multiple potential influences, it seems that the current evidence does not provide a very exact explanation (34–36), and AHA and ERC could therefore not make strong recommendations but could only offer guidance on some of these factors. Since the cooling method and temperature level did not affect the clinical outcome of CA patients in the RCT, we must conclude that it is difficult to improve the clinical outcome from either the TTM method or technology.

As we were at a loss and had to deny any benefit of TTM, we reviewed the above recent RCT again. We found that although this trial did not show a significant difference in survival and showed poor neurologic outcome at 6 months regardless of whether patients received TTM [RR = 1.04; 95% CI (0.94–1.14); *p* = 0.37 and RR = 1.00; 95% CI (0.92–1.09), respectively], the survival rate was surprisingly high in both groups [hypothermia group: 460/925 (50%); normothermia group: 479/925 (56%)] (10). in China, the survival rate of OHCA patients was ~1% in 2018 (37). Moreover, the survival rate at discharge of OHCA with ROSC was <20% in China, and these data come from the standard cardiac arrest center (38). We did not question the methodology of this RCT, and we hypothesized that, in addition to TTM, there were other factors influencing the results. Many demographic characteristics, for example, can influence results, but the cause of CA is one of the most significant factors influencing clinical outcomes for patients. A recent meta-analysis showed that patients with CA caused by acute coronary syndromes, ventricular tachycardia, ventricular fibrillation, and other heart diseases had better survival outcomes than patients with CA from other causes [OR = 3.76, 95% CI (2.95–4.78), *p* <0.001] (39). Moreover, an interesting trial showed that TTM might increase mortality for patients with non-shockable rhythm. However, this trial did not match the time of ROSC, cause of CA and other baseline characteristics, which have a significant underlying influence on the prognosis of CA (40). In summary, it was associated with typical clinical manifestations, specific laboratory results, and mature removal techniques of etiology (41).

The other most important factor influencing patients' clinical outcomes is the time of collapse to ROSC. When the time of collapse to ROSC is extended, the patient's clinical outcome will deteriorate, despite an effective and high-quality implementation of CPR. However, concepts of pathology and physiopathology can explain the observation (42–44). Therefore, patient outcomes are based on the time of collapse to ROSC, regardless of whether any intervention is applied. If an intervention is applied for patients with shorter or longer intervals of collapse to ROSC, there may be no significant clinical benefit for patients with CA. Therefore, it is too early to completely deny the role of TTM. In *in vitro* and animal trials, TTM reduced injury to cells (45). We speculate that the level of cell injury or necrosis does not cause organ dysfunction or failure within 20 min of collapse to ROSC, so although TTM lessens the degree of cell damage, it does not show an improvement in clinical outcomes. In contrast, when TTM is applied for patients with a longer interval of collapse to ROSC (≥40 min in this meta-analysis), organ function damage has already appeared and is irreversible. Based on this network meta-analysis, patients with a time from collapse to ROSC <20 min might have mild injury, and patients with a time from collapse to ROSC more than 40 min might have severe injury in organs. Because clinical outcomes in these patients may be predicted with a high probability, the role of TTM may be misestimated by studies including the above patients with shorter or longer times from collapse to ROSC. Most importantly, more attention should be given to patients with moderate injury (the time from collapse to ROSC within 20–40 min) who are at high risk of developing severe injury. Thus, just as with primary percutaneous coronary intervention or emergency thrombolysis for acute myocardial infarction or ischemic stroke, there might be an optimal time window to intervene for improving clinical outcomes for patients with CA (41, 46). If the results we reach may be confirmed in the future, we might be able to put an end to endless debate about TTM. In other words, avoiding ineffective application of TTM can not only reduce the cost burden but also avoid the occurrence of TTM-related adverse events, such as bleeding or bradyarrhythmia. Based upon time from collapse to ROSC, successful trials may be achieved by excluding patients who will become well regardless of what and those who are likely to become poorly regardless of what. Based on the above evidence and speculation, we conducted this network meta-analysis, and we did find an optimal time window, within 20–39 min of collapse to ROSC, in which to apply TTM. Moreover, we believe that the optimal time window may not only benefit patients but also provide a meaningful reference for future trials. Of course, TTM is only one of the most well-established interventions. There are certainly other interventions that are effective in improving patients' clinical outcomes, and it only remains for us to find evidence to support their application.

## Limitations

This network meta-analysis has the following limitations: (a) since most of the included trials were retrospective, we could only cautiously summarize their conclusions; (b) we did not utilize a subgroup with which to compare clinical outcomes among different temperature levels; (c) the number of patients and trials was small, and might have biased the results; (d) both in survival and neurologic outcome, due to the lack of direct comparison between some of the groups, a complete closed loop could not be formed; there might therefore be some bias in the results of only indirect comparisons; (e) the 20-min interval might be too broad, and thus may mask some of the underlying factors; (f) we only compared short-term (30 days and at discharge) clinical outcomes.

## Conclusion

From this network meta-analysis, we drew the following conclusions: (a) the survival and neurologic outcomes were related to the time of collapse to ROSC, and with the extension of time, these clinical outcomes would deteriorate for patients with CA; (b) TTM is still effective for CA patients to improve short-term clinical outcomes, however, this effect might be shown only within 20–40 min of collapse to ROSC; (c) the effectiveness of TTM might be inconclusive in improving survival, but its role in improving neurologic outcome should be recognized. Of course, based on our findings, further trials should pay more attention to patients with moderate time (20–39 min) from collapse to ROSC who suffer from moderate injury and intend to become worse.

## Data Availability Statement

The original contributions presented in the study are included in the article/Supplementary Material, further inquiries can be directed to the corresponding author/s.

## Author Contributions

JD: writing, searching, and statistics. QZ: draw pictures and table and collection. YS: screening articles. HG: screening articles and editing language. KZ: language editing and providing sensitive analysis. LD and BD: methodology. JY: methodology and editing language. QM: conceptualization and methodology. All authors contributed to the article and approved the submitted version.

## Conflict of Interest

The authors declare that the research was conducted in the absence of any commercial or financial relationships that could be construed as a potential conflict of interest.

## Publisher's Note

All claims expressed in this article are solely those of the authors and do not necessarily represent those of their affiliated organizations, or those of the publisher, the editors and the reviewers. Any product that may be evaluated in this article, or claim that may be made by its manufacturer, is not guaranteed or endorsed by the publisher.
