# Supplementary material for: Optimal Time of Collapse to Return of Spontaneous Circulation to Apply Targeted Temperature Management for Cardiac Arrest: A Bayesian Network Meta-Analysis
Source: Front Cardiovasc Med. 2022 Jan 7;8:784917. doi: 10.3389/fcvm.2021.784917 (PMC8777010; doi:10.3389/fcvm.2021.784917)
Supplement: Supplementary file 1 [file Data_Sheet_1.docx]

**Search strategy**

**PubMed:**

("Cardiopulmonary Resuscitation"[Mesh] or Resuscitation, Cardiopulmonary or CPR or Cardio-Pulmonary Resuscitation or Cardio Pulmonary Resuscitation or Resuscitation, Cardio-Pulmonary or Code Blue or Mouth-to-Mouth Resuscitation or Mouth to Mouth Resuscitation or Mouth-to-Mouth Resuscitations or Resuscitation, Mouth-to-Mouth or Resuscitations, Mouth-to-Mouth or Basic Cardiac Life Support or Life Support, Basic Cardiac) and ("Hypothermia, Induced"[Mesh] or Therapeutic Hypothermia or Hypothermia, Therapeutic or Targeted Temperature Management or Targeted Temperature Managements or Induced Hypothermia or Moderate Hypothermia, Induced or Induced Moderate Hypothermia or Induced Moderate Hypothermias or Moderate Hypothermias, Induced or Mild Hypothermia, Induced or Induced Mild Hypothermia or Induced Mild Hypothermias or Mild Hypothermias, Induced) and ("Heart Arrest"[Mesh] or Arrest, Heart or Cardiac Arrest or Arrest, Cardiac or Asystole or Asystoles or Cardiopulmonary Arrest or Arrest, Cardiopulmonary or ((pulseless* or nonperfus*) and (ventricular tachycardia* or ventricular tachyarrhythmia)) or ventricular fibrillation* or Pulseless electrical activity or PEA)

**Embase:**

**#**1 Cardiopulmonary Resuscitation/exp

#2 Resuscitation, Cardiopulmonary

#3 CPR

#4 Cardio-Pulmonary Resuscitation

#5 Code Blue

#6 Mouth-to-Mouth Resuscitation*

#7 Mouth-to-Mouth

#8 Basic Cardiac Life Support

#9 Life Support, Basic Cardiac

#10 #1 or #2 or #3 or #4 or #5 or #6 or #7 or #8 or #9

#11 Hypothermia, Induced

#12 Therapeutic Hypothermia/exp

#13 Targeted Temperature Management*

#14 TTM

#15 Induced Hypothermia

#16 Moderate Hypothermia, Induced

#17 Induced Moderate Hypothermia*

#18 Induced Mild Hypothermia*

#19 #11 or #12 or #13 or #14 or #15 or #16 or #17 or #18

#20 Heart Arrest/exp

#21 Cardiac Arrest

#22 Asystole*

#23 Cardiopulmonary Arrest

#24 Ventricular fibrillation*

#25 Pulseless electrical activity

#26 PEA

#27 #20 or #21 or #22 or #23 or #24 or #25 or #26

#28 Pulseless*

#29 Nonperfus*

#30 Ventricular tachycardia*

#31 Ventricular tachyarrhythmia

#32 #28 or #29

#33 #30 or #31

#34 #32 and #33

#35 #27 or #34

#36 #10 and #19 and #35

**CENTRAL:**

**#**1 MeSH descriptor: [Cardiopulmonary resuscitation] explode all trees

#2 Resuscitation, Cardiopulmonary

#3 CPR

#4 Cardio-Pulmonary Resuscitation

#5 Code Blue

#6 Mouth-to-Mouth Resuscitation*

#7 Mouth-to-Mouth

#8 Basic Cardiac Life Support

#9 Life Support, Basic Cardiac

#10 #1 or #2 or #3 or #4 or #5 or #6 or #7 or #8 or #9

#11 MeSH descriptor: [Hypothermia, Induced] explode all trees

#12 Therapeutic Hypothermia

#13 MeSH descriptor: [Targeted Temperature Management] explode all trees

#14 TTM

#15 Induced Hypothermia

#16 Moderate Hypothermia, Induced

#17 Induced Moderate Hypothermia*

#18 Induced Mild Hypothermia*

#19 #11 or #12 or #13 or #14 or #15 or #16 or #17 or #18

#20 MeSH descriptor: [Heart Arrest] explode all trees

#21 Cardiac Arrest

#22 MeSH descriptor: [Asystole] explode all trees

#23 Asystole*

#24 Cardiopulmonary Arrest

#25 MeSH descriptor: [Ventricular fibrillation] explode all trees

#26 Pulseless electrical activity

#27 PEA

#28 #20 or #21 or #22 or #23 or #24 or #25 or #26 or #27

#29 Pulseless*

#30 Nonperfus*

#31 MeSH descriptor: [Ventricular tachycardia] explode all trees

#32 Ventricular tachyarrhythmia

#33 #29 or #30

#34 #31 or #32

#35 #33 and #34

#36 #28 or #35

#37 #10 and #19 and #36

**Web of science:**

#1 all=(Cardiopulmonary Resuscitation or Resuscitation, Cardiopulmonary or CPR or Cardio-Pulmonary Resuscitation or Cardio Pulmonary Resuscitation or Resuscitation, Cardio-Pulmonary or Code Blue or Mouth-to-Mouth Resuscitation or Mouth to Mouth Resuscitation or Mouth-to-Mouth Resuscitations or Resuscitation, Mouth-to-Mouth or Resuscitations, Mouth-to-Mouth or Basic Cardiac Life Support or Life Support, Basic Cardiac)

#2 all=(Hypothermia, Induced or Therapeutic Hypothermia or Hypothermia, Therapeutic or Targeted Temperature Management or Targeted Temperature Managements or Induced Hypothermia or Moderate Hypothermia, Induced or Induced Moderate Hypothermia or Induced Moderate Hypothermias or Moderate Hypothermias, Induced or Mild Hypothermia, Induced or Induced Mild Hypothermia or Induced Mild Hypothermias or Mild Hypothermias, Induced)

#3 all=(Heart Arrest or Arrest, Heart or Cardiac Arrest or Arrest, Cardiac or Asystole or Asystoles or Cardiopulmonary Arrest or Arrest, Cardiopulmonary or ((pulseless* or nonperfus*) and (ventricular tachycardia* or ventricular tachyarrhythmia)) or ventricular fibrillation* or Pulseless electrical activity or PEA)

#4 #1 AND #2 AND #3

**Supplementary materials**

|  | Group | | | | | | | |
| --- | --- | --- | --- | --- | --- | --- | --- | --- |
| Rank | a | b | c | d | e | f | g | h |
| Best | 46.1 | 43.1 | 6.4 | 0.0 | 0.7 | 0.0 | 3.5 | 0.0 |
| 2nd | 33.2 | 42.1 | 20.0 | 0.4 | 1.6 | 0.1 | 2.6 | 0.0 |
| 3rd | 11.5 | 15.5 | 60.6 | 2.6 | 5.4 | 0.3 | 4.1 | 0.0 |
| 4th | 2.5 | 3.9 | 11.1 | 46.4 | 24.8 | 2.3 | 8.8 | 0.2 |
| 5th | 0.6 | 1.0 | 1.8 | 31.4 | 44.0 | 10.3 | 10.4 | 0.4 |
| 6th | 0.1 | 0.2 | 0.1 | 15.7 | 19.9 | 44.7 | 17.5 | 1.8 |
| 7th | 0.0 | 0.1 | 0.0 | 3.4 | 3.4 | 38.4 | 45.3 | 9.5 |
| Worst | 0.0 | 0.0 | 0.0 | 0.1 | 0.2 | 3.9 | 7.8 | 88.1 |

**Supplementary table 1.** Rank probability of survival (a:<20min+TTM; b:<20min; c:20-39min+TTM; d:20-39min+TTM; e:20-39min; f:40-59min+TTM; g:≥60min+TTM; h:≥60min)

**
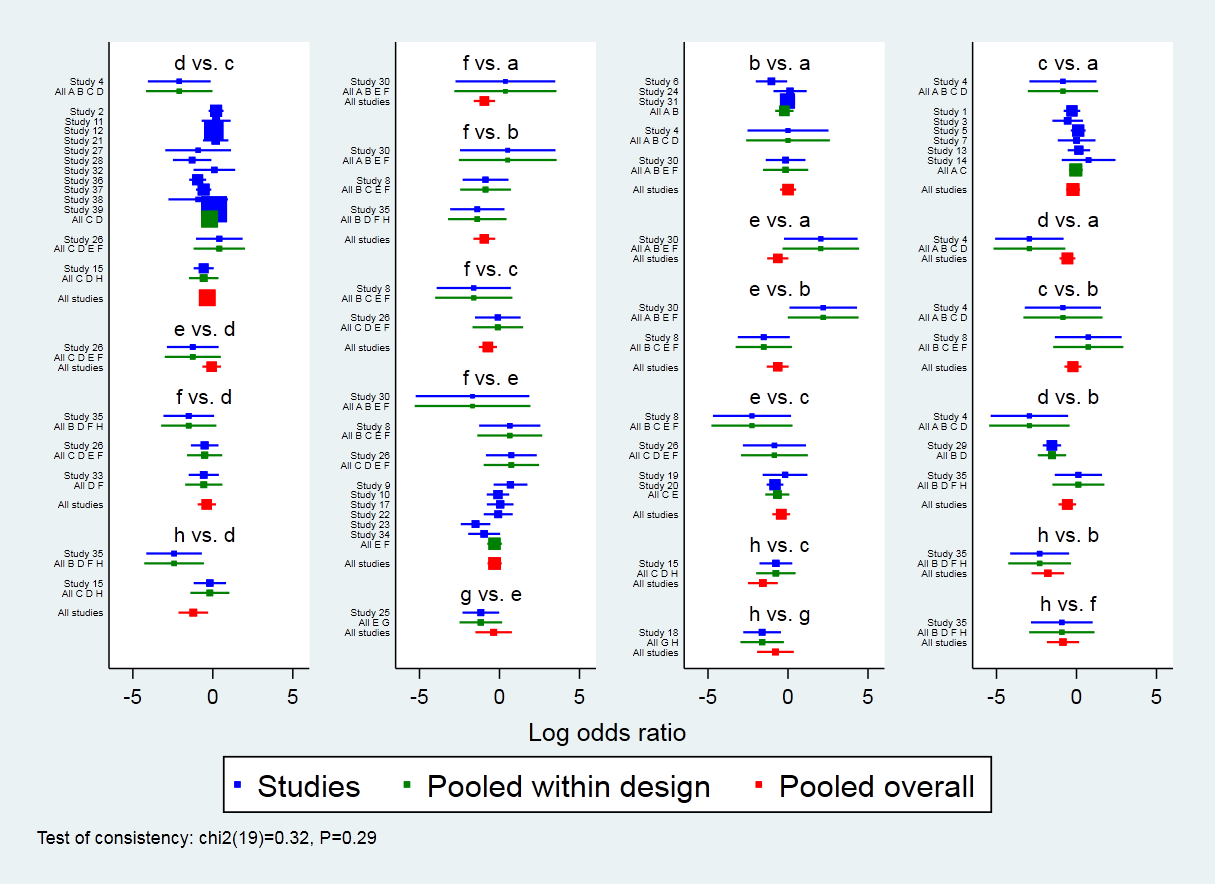
**

**Supplementary figure 1.** forest plot for global inconsistency of survival (a:<20min+TTM; b:<20min; c:20-39min+TTM; d:20-39min+TTM; e:20-39min; f:40-59min+TTM; g:≥60min+TTM; h:≥60min)

**
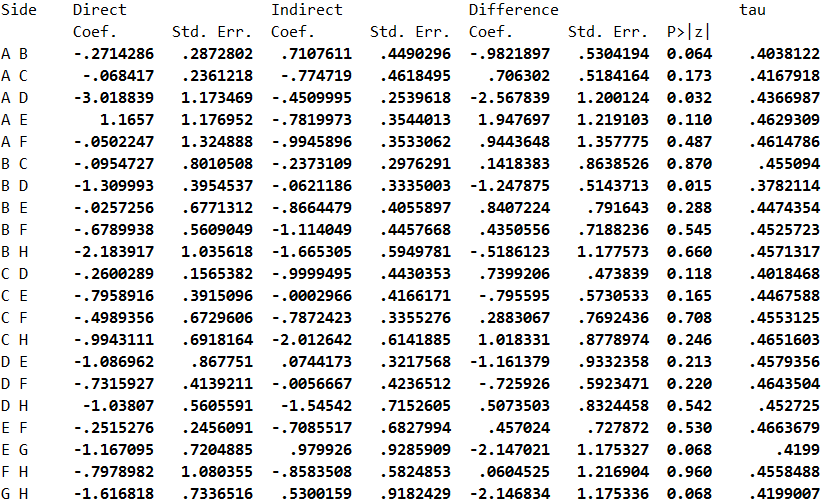
Supplementary figure 2.** side split test plot for survival (A:<20min+TTM; B:<20min; C:20-39min+TTM; D:20-39min+TTM; E:20-39min; F:40-59min+TTM; G:≥60min+TTM; H:≥60min. If p>0.5, between the two groups have local inconsistency)

| Loop | IF | seIF | Z value | P value | 95% CI | Loop_Heterog_tau2 |
| --- | --- | --- | --- | --- | --- | --- |
| A-D-E | 6.251 | 1.802 | 3.470 | 0.001 | (2.72,9.78) | 0.000 |
| A-D-F | 3.983 | 1.953 | 2.039 | 0.041 | (0.16,7.81) | 0.000 |
| D-E-G-H | 3.285 | 1.258 | 2.611 | 0.009 | (0.82,5.75) | 0.000 |
| A-C-E | 2.912 | 1.205 | 2.417 | 0.016 | (0.55,5.27) | 0.000 |
| C-E-G-H | 2.825 | 1.018 | 2.775 | 0.006 | (0.83,4.82) | 0.000 |
| B-D-E | 2.767 | 2.560 | 1.081 | 0.280 | (0.00,7.78) | 1.963 |
| A-C-D | 2.680 | 1.145 | 2.341 | 0.019 | (0.44,4.92) | 0.067 |
| A-B-E | 2.276 | 2.036 | 1.118 | 0.264 | (0.00,6.27) | 0.487 |
| E-F-G-H | 1.659 | 1.619 | 1.025 | 0.305 | (0.00,4.83) | 0.279 |
| B-C-H | 1.595 | 1.342 | 1.189 | 0.234 | (0.00,4.22) | 0.000 |
| A-E-F | 1.509 | 2.183 | 0.691 | 0.489 | (0.00,5.79) | 0.295 |
| B-D-F | 1.462 | 1.047 | 1.397 | 0.162 | (0.00,3.51) | 0.237 |
| A-B-D | 1.425 | 1.442 | 0.989 | 0.323 | (0.00,4.25) | 0.221 |
| A-B-F | 1.379 | 1.683 | 0.820 | 0.412 | (0.00,4.68) | 0.001 |
| B-C-D | 1.144 | 1.001 | 1.143 | 0.253 | (0.00,3.11) | 0.110 |
| A-C-F | 0.947 | 1.713 | 0.553 | 0.580 | (0.00,4.30) | 0.000 |
| D-E-F | 0.813 | 1.058 | 0.769 | 0.442 | (0.00,2.89) | 0.197 |
| B-F-H | 0.775 | 1.511 | 0.513 | 0.608 | (0.00,3.74) | 0.000 |
| C-D-E | 0.715 | 1.003 | 0.713 | 0.476 | (0.00,2.68) | 0.105 |
| D-F-H | 0.700 | 1.128 | 0.621 | 0.535 | (0.00,2.91) | 0.000 |
| B-C-E | 0.696 | 1.663 | 0.418 | 0.676 | (0.00,3.96) | 0.740 |
| C-F-H | 0.679 | 1.275 | 0.532 | 0.594 | (0.00,3.18) | 0.000 |
| B-E-F | 0.648 | 1.232 | 0.526 | 0.599 | (0.00,3.06) | 0.474 |
| B-E-G-H | 0.612 | 1.421 | 0.431 | 0.667 | (0.00,3.40) | 0.000 |
| C-E-F | 0.568 | 0.855 | 0.665 | 0.506 | (0.00,2.24) | 0.205 |
| C-D-F | 0.413 | 0.814 | 0.508 | 0.612 | (0.00,2.01) | 0.105 |
| B-C-F | 0.364 | 1.347 | 0.270 | 0.787 | (0.00,3.00) | 0.128 |
| C-D-H | 0.324 | 0.968 | 0.335 | 0.738 | (0.00,2.22) | 0.127 |
| B-D-H | 0.057 | 1.078 | 0.053 | 0.958 | (0.00,2.17) | 0.000 |
| A-B-C | 0.022 | 0.828 | 0.026 | 0.979 | (0.00,1.64) | 0.000 |

**Supplementary table 2.** loop inconsistency for survival (A:<20min+TTM; B:<20min; C:20-39min+TTM; D:20-39min+TTM; E:20-39min; F:40-59min+TTM; G:≥60min+TTM; H:≥60min. If 95% confidence interval contains 1, the loop inconsistency is not significant)

**
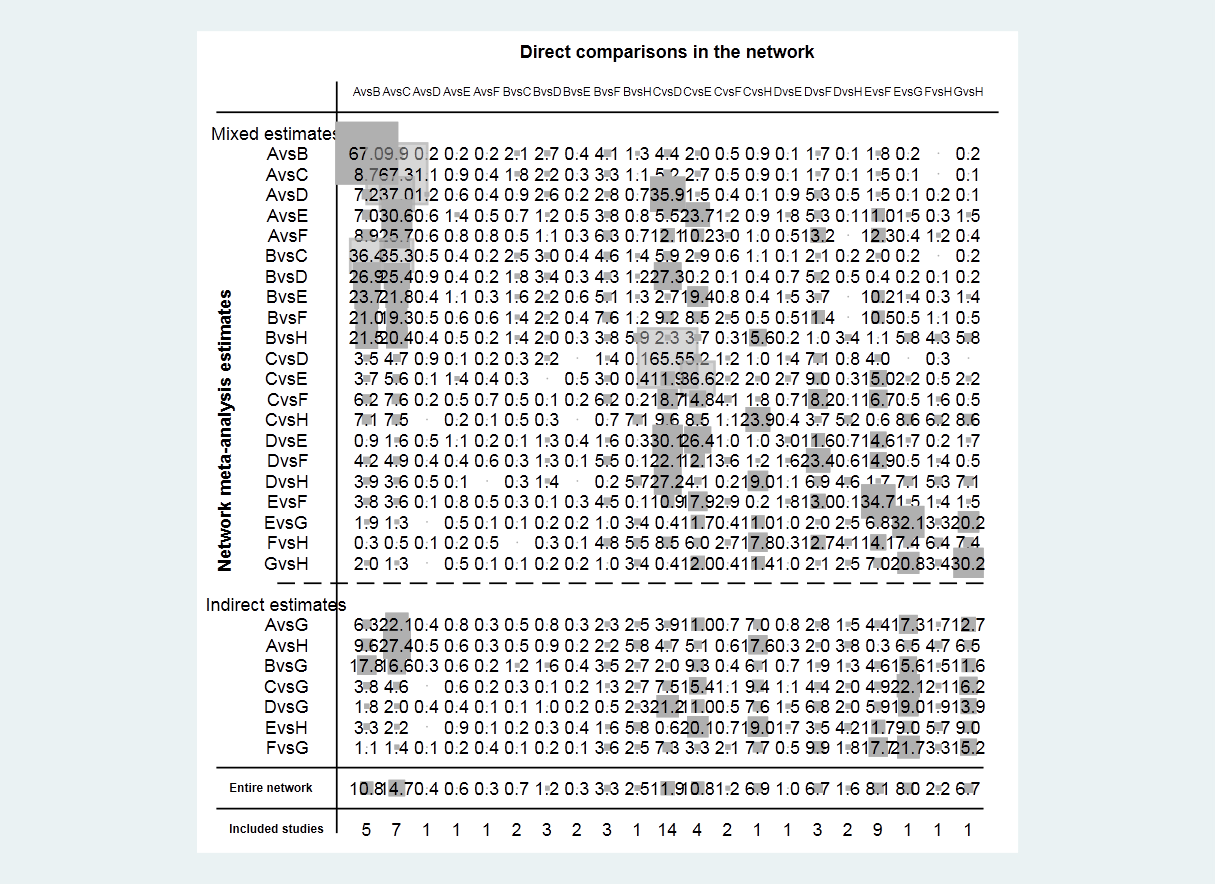
**

**Supplementary figure 3.**  Netweight plot for survival (A:<20min+TTM; B:<20min; C:20-39min+TTM; D:20-39min+TTM; E:20-39min; F:40-59min+TTM; G:≥60min+TTM; H: ≥60min. This plot shows the weight of indirect and direct comparison)

**
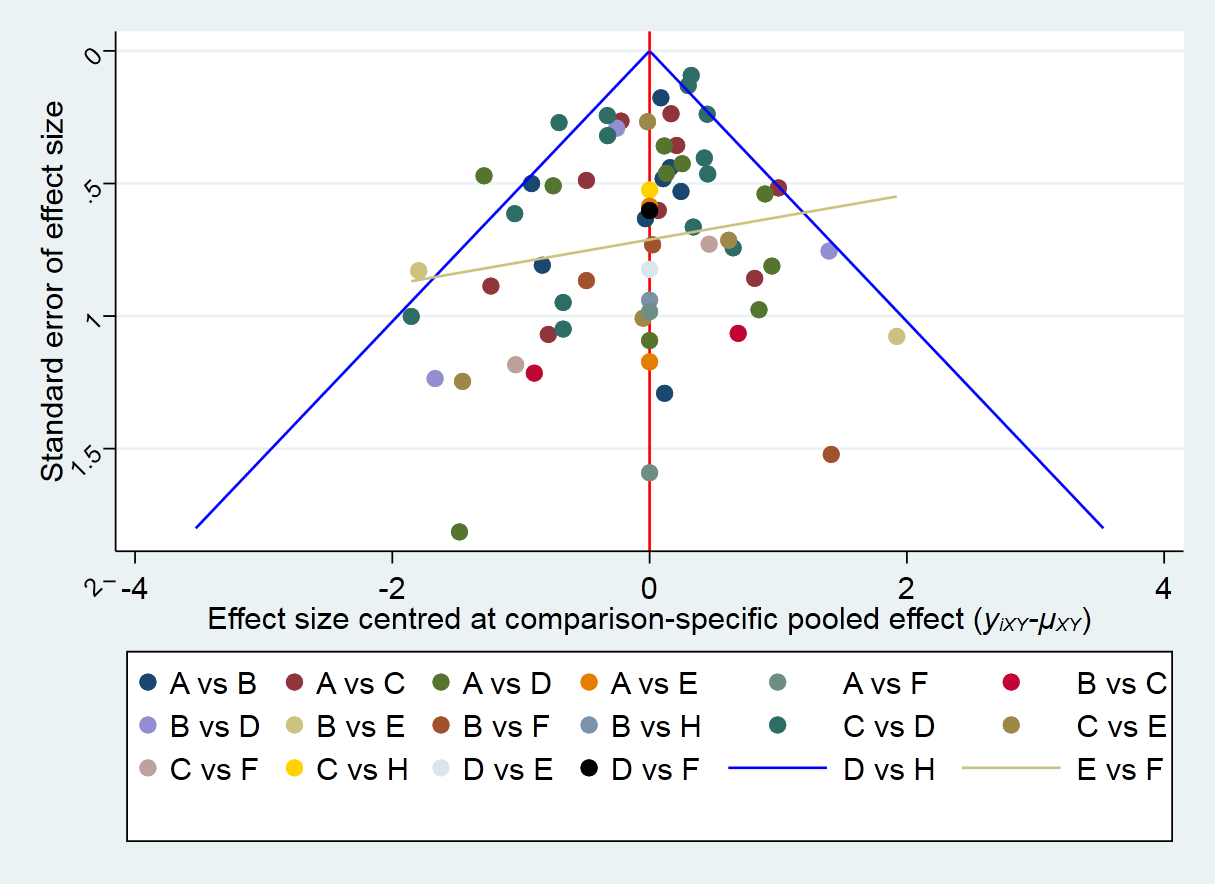
**

**Supplementary figure 4.** Funnel plot for survival (A:<20min+TTM; B:<20min; C:20-39min+TTM; D:20-39min+TTM; E:20-39min; F:40-59min+TTM; G:≥60min+TTM; H: ≥60min)

|  | Treatment | | | | | | | |
| --- | --- | --- | --- | --- | --- | --- | --- | --- |
| Rank | a | b | c | d | e | f | g | h |
| Best | 52.5 | 42.6 | 4.7 | 0.0 | 0.1 | 0.0 | 0.1 | 0.0 |
| 2nd | 41.1 | 37.4 | 21.0 | 0.2 | 0.3 | 0.0 | 0.1 | 0.0 |
| 3rd | 5.9 | 17.9 | 72.2 | 2.8 | 0.9 | 0.0 | 0.3 | 0.0 |
| 4th | 0.5 | 2.0 | 2.1 | 81.9 | 12.7 | 0.4 | 0.4 | 0.0 |
| 5th | 0.0 | 0.1 | 0.1 | 13.6 | 73.2 | 11.7 | 1.2 | 0.1 |
| 6th | 0.0 | 0.0 | 0.0 | 1.4 | 12.6 | 81.3 | 4.4 | 0.3 |
| 7th | 0.0 | 0.0 | 0.0 | 0.0 | 0.3 | 6.0 | 90.1 | 3.6 |
| Worst | 0.0 | 0.0 | 0.0 | 0.0 | 0.0 | 0.5 | 3.5 | 96.0 |

**Supplementary table 3: Rank probability of good neurologic outcome**

**
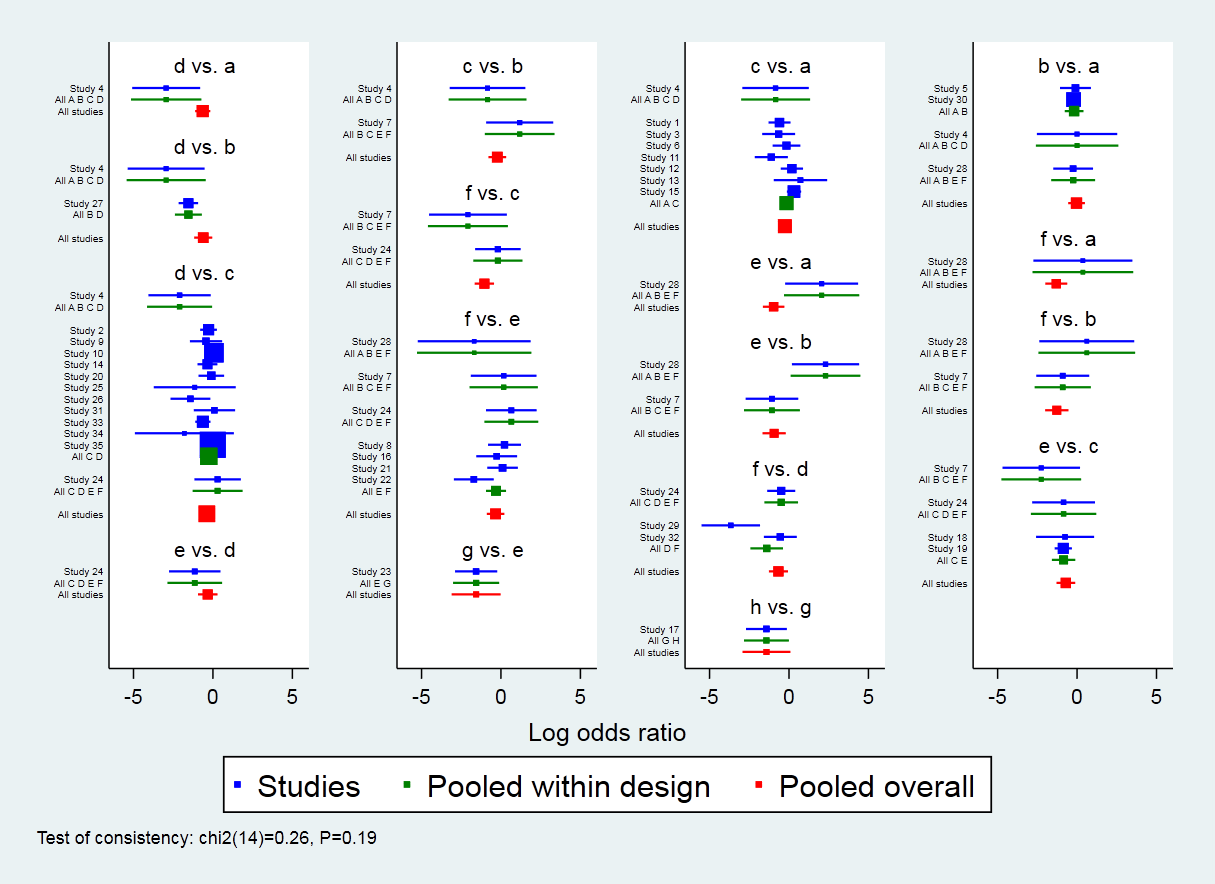
**

**Supplementary figure 5.** Forest plot for global inconsistency of good neurologic outcome (a:<20min+TTM; b:<20min; c:20-39min+TTM; d:20-39min+TTM; e:20-39min; f:40-59min+TTM; g:≥60min+TTM; h:≥60min)

**
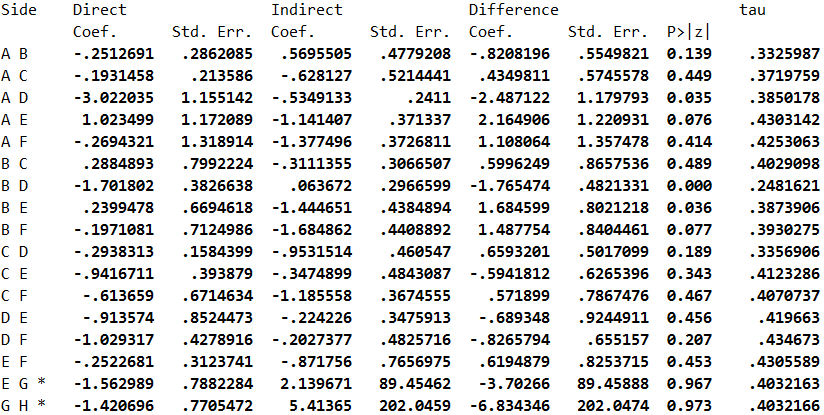
**

**Supplementary figure 6.** side split test plot for good neurologic outcome (A:<20min+TTM; B:<20min; C:20-39min+TTM; D:20-39min+TTM; E:20-39min; F:40-59min+TTM; G:≥60min+TTM; H:≥60min. If p>0.5, between the two groups have local inconsistency)

| Loop | IF | seIF | Z value | P value | 95% CI | Loop_Heterog_tau2 |
| --- | --- | --- | --- | --- | --- | --- |
| A-D-E | 6.142 | 1.803 | 3.407 | 0.001 | (2.61,9.68) | 0.000 |
| A-D-F | 4.661 | 3.372 | 1.382 | 0.167 | (0.00,11.27) | 1.403 |
| B-D-E | 3.722 | 2.505 | 1.486 | 0.137 | (0.00,8.63) | 2.287 |
| A-C-E | 3.167 | 1.262 | 2.510 | 0.012 | (0.69,5.64) | 0.074 |
| B-D-F | 2.901 | 1.714 | 1.692 | 0.091 | (0.00,6.26) | 1.046 |
| A-C-D | 2.584 | 1.138 | 2.271 | 0.023 | (0.35,4.81) | 0.047 |
| A-B-E | 1.892 | 1.944 | 0.973 | 0.331 | (0.00,5.70) | 0.338 |
| B-C-D | 1.746 | 0.894 | 1.953 | 0.051 | (0.00,3.50) | 0.030 |
| A-E-F | 1.513 | 2.116 | 0.715 | 0.474 | (0.00,5.66) | 0.230 |
| A-C-F | 1.299 | 1.791 | 0.726 | 0.468 | (0.00,4.81) | 0.145 |
| A-B-D | 1.109 | 1.146 | 0.968 | 0.333 | (0.00,3.36) | 0.000 |
| A-B-F | 1.107 | 1.764 | 0.628 | 0.530 | (0.00,4.57) | 0.000 |
| B-C-E | 0.959 | 1.571 | 0.611 | 0.541 | (0.00,4.04) | 0.640 |
| B-C-F | 0.632 | 1.319 | 0.479 | 0.632 | (0.00,3.22) | 0.000 |
| C-E-F | 0.599 | 0.849 | 0.706 | 0.480 | (0.00,2.26) | 0.126 |
| B-E-F | 0.588 | 1.508 | 0.390 | 0.697 | (0.00,3.54) | 0.670 |
| C-D-F | 0.565 | 0.942 | 0.600 | 0.548 | (0.00,2.41) | 0.102 |
| C-D-E | 0.447 | 0.906 | 0.493 | 0.622 | (0.00,2.22) | 0.037 |
| D-E-F | 0.300 | 1.460 | 0.205 | 0.837 | (0.00,3.16) | 0.626 |
| A-B-C | 0.234 | 0.881 | 0.265 | 0.791 | (0.00,1.96) | 0.062 |

**Supplementary table 4.** loop inconsistency for good neurologic outcome (A:<20min+TTM; B:<20min; C:20-39min+TTM; D:20-39min+TTM; E:20-39min; F:40-59min+TTM; G:≥60min+TTM; H:≥60min. If 95% confidence interval contains 1, the loop inconsistency is not significant)


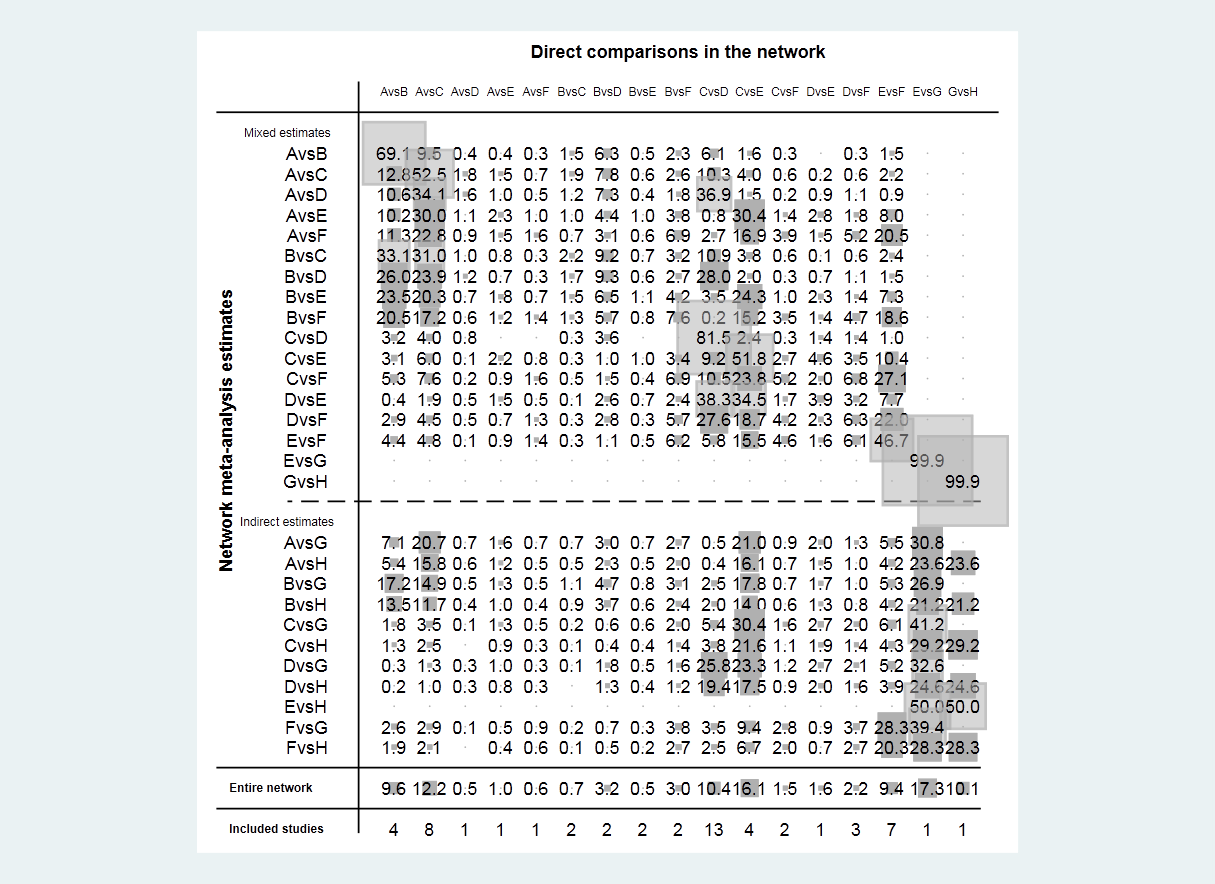


**Supplementary figure 7.**  Netweight plot for good neurologic outcome (A:<20min+TTM; B:<20min; C:20-39min+TTM; D:20-39min+TTM; E:20-39min; F:40-59min+TTM; G:≥60min+TTM. This plot shows the weight of indirect and direct comparison)


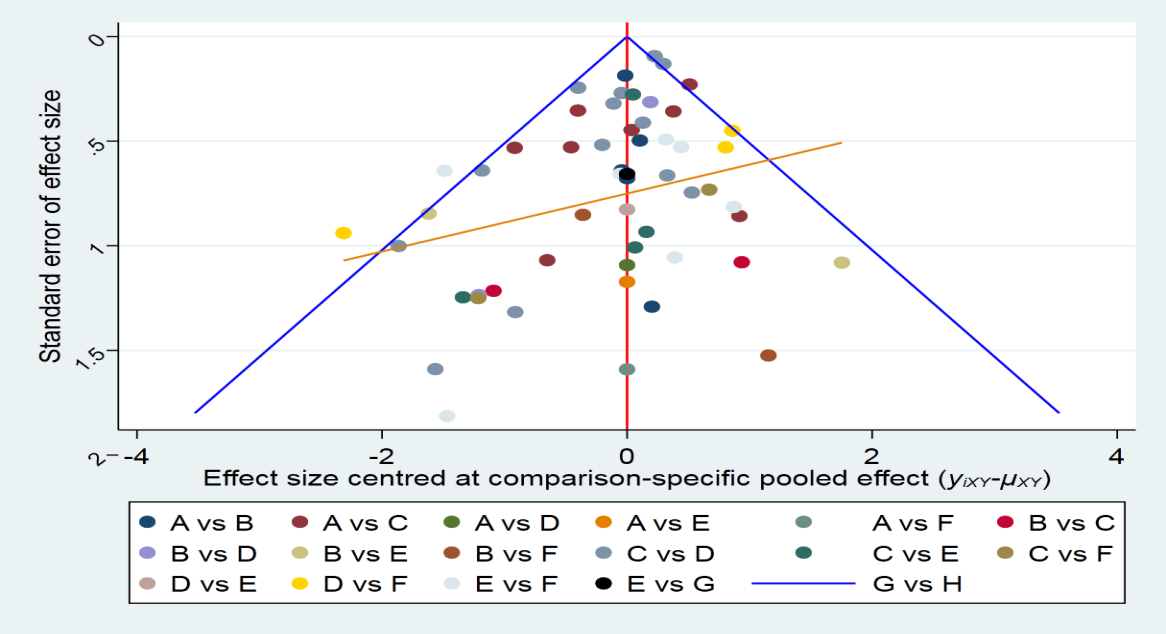


**Supplementary figure 8.** Funnel plot for good neurologic outcome (A:<20min+TTM; B:<20min; C:20-39min+TTM; D:20-39min+TTM; E:20-39min; F:40-59min+TTM; G:≥60min+TTM; H: ≥60min)

**
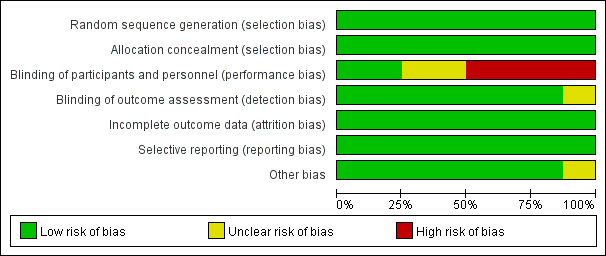
**

**Supplementary figure 9.** Risk of bias graph for randomized controlled trials

**
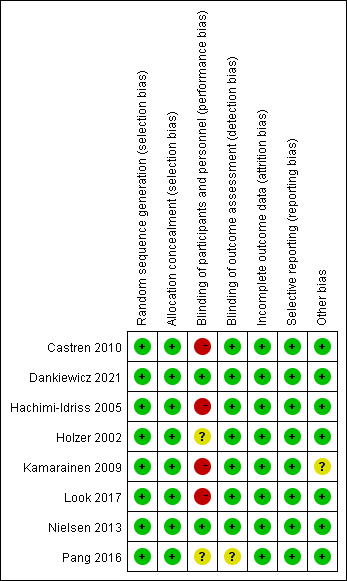
**

**Supplementary Figure 10.** Risk of bias summary for randomized controlled trials

| Author name | Selection | Comparability | exposure | NOS score |
| --- | --- | --- | --- | --- |
| Agarwal et al. | 3 | 2 | 3 | 8 |
| Arrich et al. | 2 | 2 | 3 | 7 |
| Blumenstein et al. | 2 | 2 | 3 | 7 |
| Chen et al. | 3 | 1 | 2 | 6 |
| Chou et al. | 3 | 2 | 3 | 8 |
| De Fazio et al. | 2 | 2 | 2 | 6 |
| Ferreira et al. | 3 | 2 | 3 | 8 |
| Fink et al. | 2 | 2 | 3 | 7 |
| Fjølner et al. | 3 | 2 | 3 | 8 |
| Gillies et al | 3 | 2 | 3 | 8 |
| Goto et al. | 3 | 2 | 2 | 7 |
| Han et al. | 3 | 3 | 2 | 8 |
| Jouffroy et al. | 2 | 2 | 2 | 6 |
| Kagawa et al. | 3 | 2 | 3 | 8 |
| Kagawa et al. | 3 | 3 | 2 | 8 |
| Kagawa et al. | 3 | 2 | 2 | 7 |
| Kim2007 et al. | 3 | 2 | 3 | 8 |
| Kim2014 et al. | 3 | 3 | 3 | 9 |
| Kim2018 et al. | 3 | 3 | 2 | 8 |
| Maekawa et al. | 3 | 2 | 3 | 8 |
| Mecklenburg et al. | 3 | 2 | 3 | 8 |
| Nagao et al. | 2 | 2 | 1 | 5 |
| Okada et al. | 3 | 2 | 2 | 7 |
| Otani et al. | 3 | 3 | 3 | 9 |
| Pang2017 et al. | 3 | 3 | 3 | 9 |
| Ryu et al. | 2 | 2 | 2 | 6 |
| Schenfeld et al. | 3 | 3 | 3 | 9 |
| Sonder et al | 3 | 2 | 2 | 7 |
| Yukawa et al. | 3 | 3 | 1 | 7 |

**Supplementary** **Table 5.** Risk of bias of each retrospective trial was assessed with the NEWCASTLE-OTTAWA QUALITY ASSESSMENT SCALE CASE CONTROL STUDIES

| Study name | Selection | Comparability | Outcome | NOS score |
| --- | --- | --- | --- | --- |
| Choi et al. | 4 | 1 | 3 | 9 |
| Schober et al. | 3 | 2 | 2 | 8 |
| Shin et al. | 3 | 2 | 2 | 7 |
| Tømte et al. | 3 | 2 | 3 | 8 |

**Supplementary** **Table 6.** Risk of bias of each cohort trial was assessed with the NEWCASTLE-OTTAWA ASSESSMENT SCALE COHORT STUDIES^[1-42]^


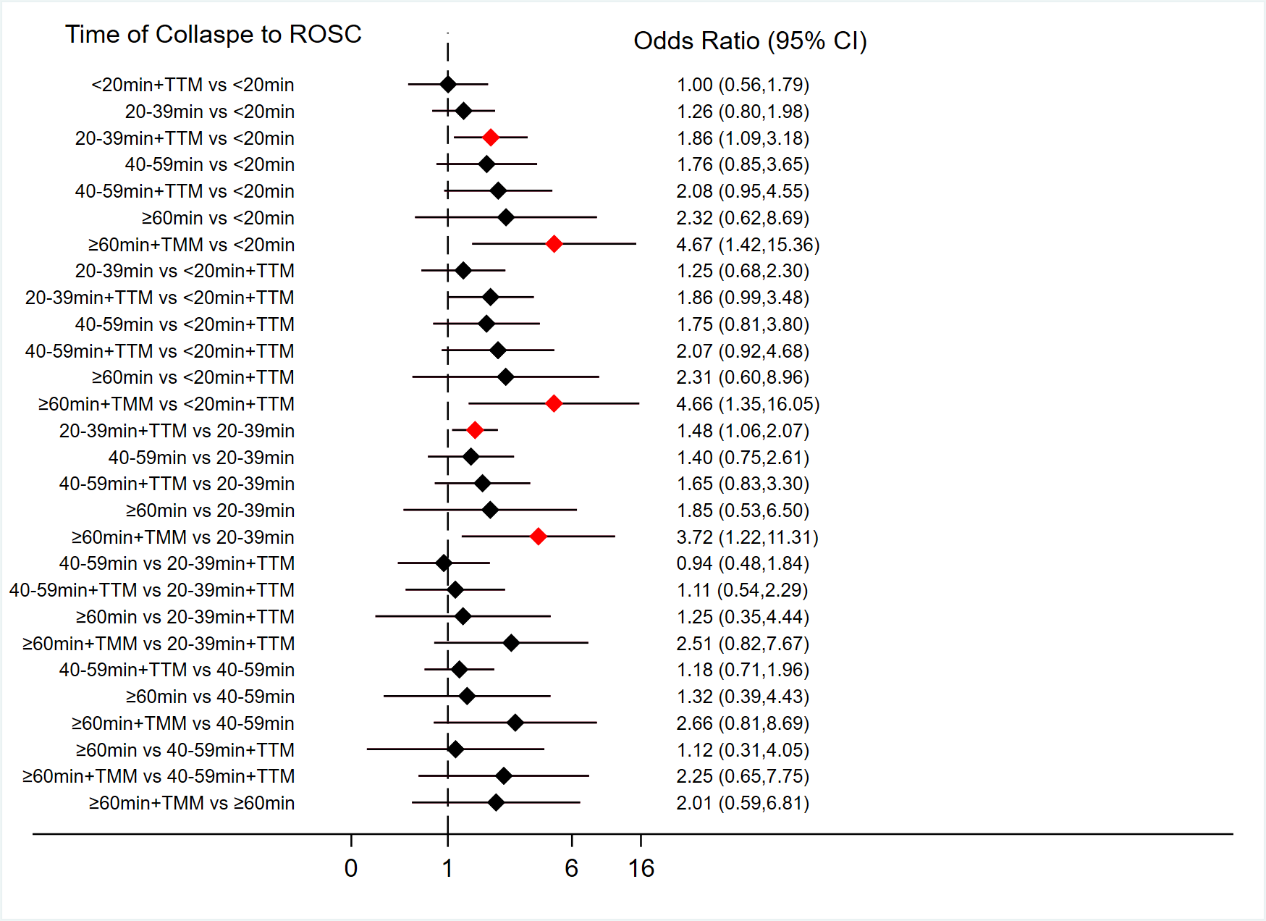


**Supplementary Figure 11.** Sensitive analysis forest plot for survival of excluding trials without OHCA, OHCA less than 10% or without record.


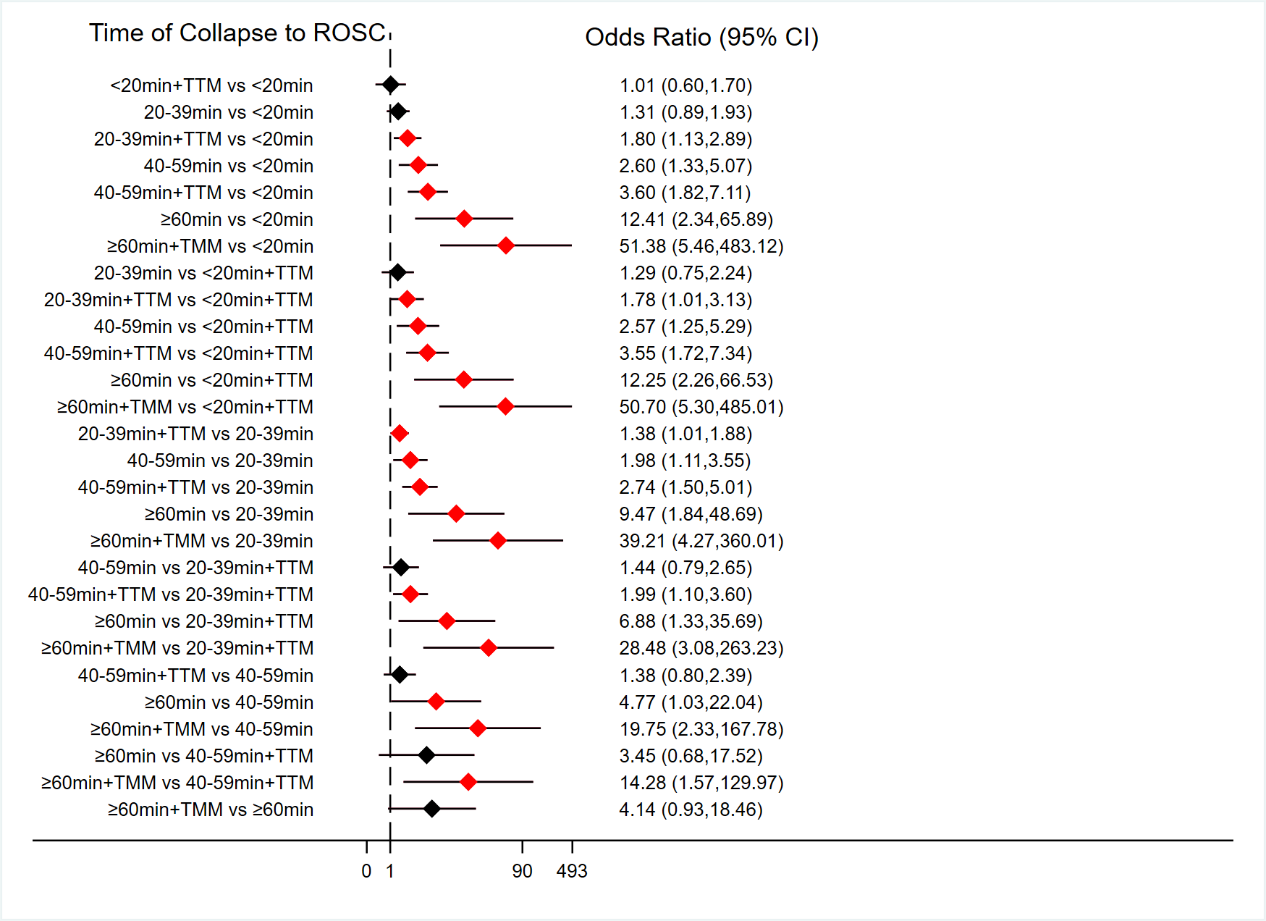


**Supplementary Figure 12.** Sensitive analysis forest plot for good neurologic outcome of excluding trials without OHCA, OHCA less than 10% or without record.


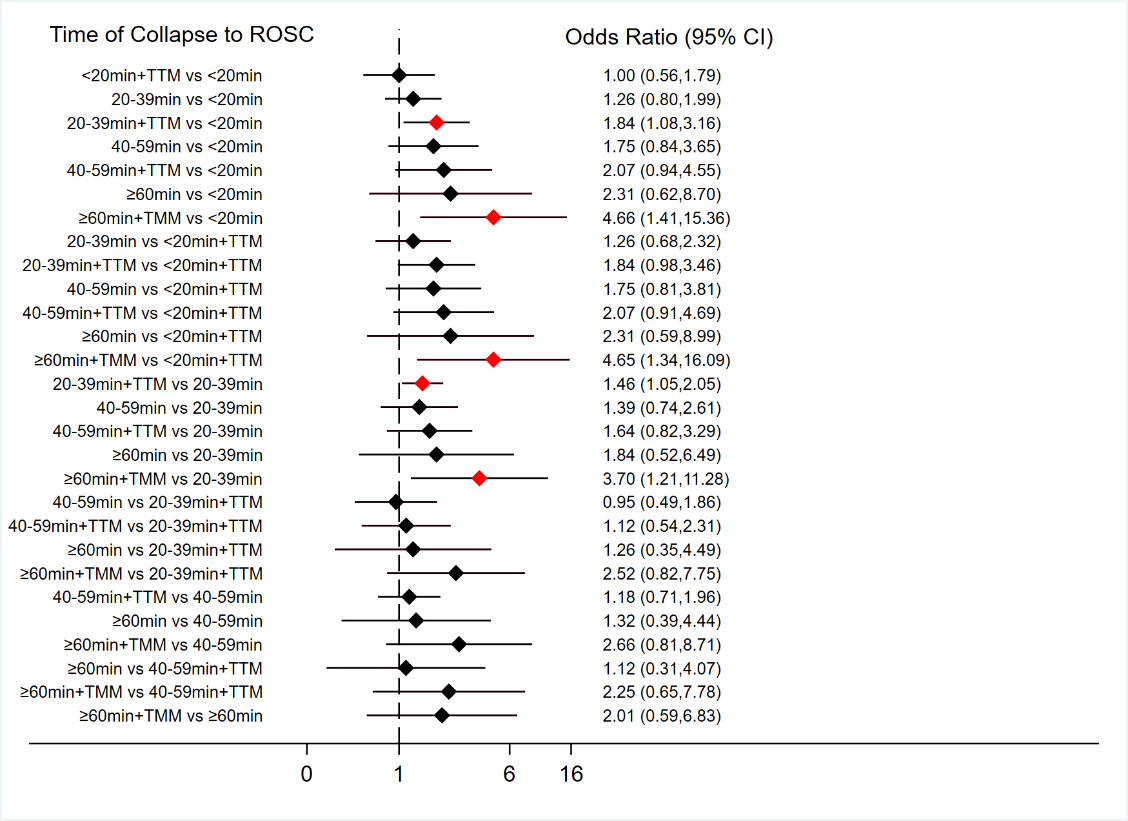


**Supplementary Figure 13.** Sensitive analysis forest plot for survival of excluding trials only including acute coronary syndromes caused cardiac arrest or without recording cause of CA which did not declare in trial or protocol.


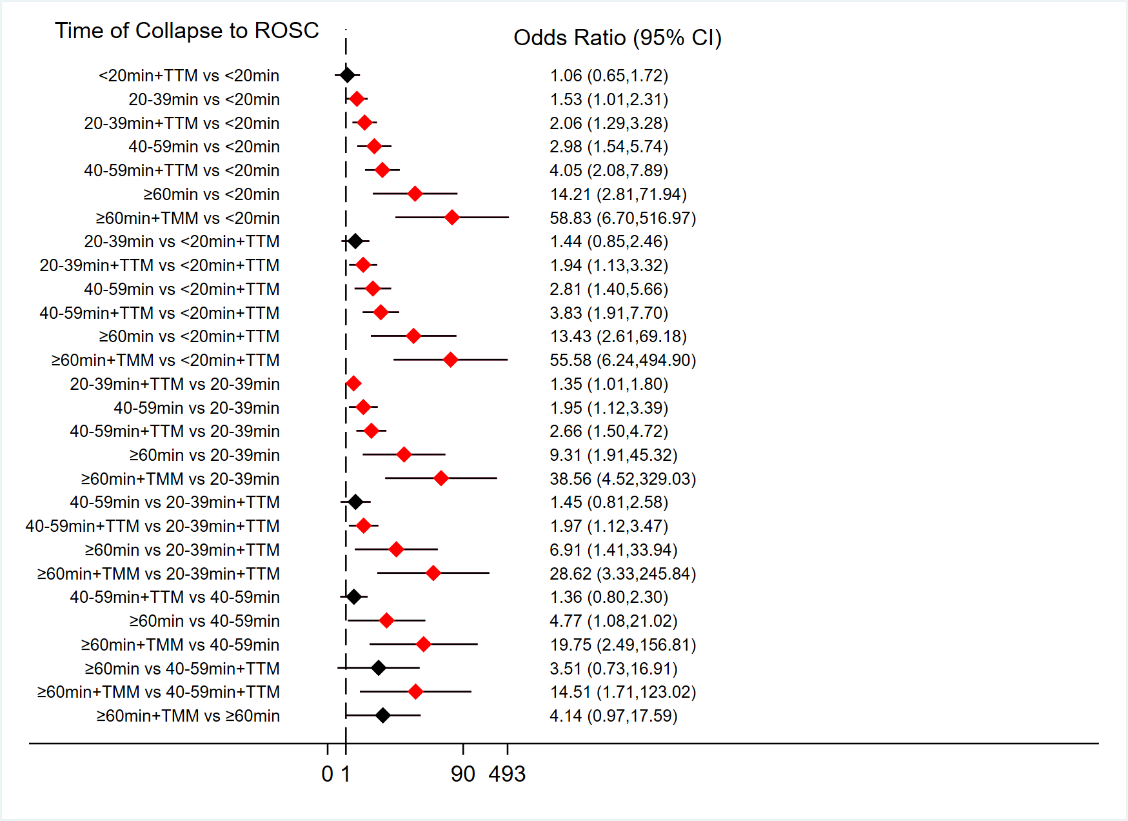


**Supplementary Figure 14.** Sensitive analysis forest plot for survival of excluding trials only including acute coronary syndromes caused cardiac arrest or without recording cause of CA which did not declare in trial or protocol.

**Reference of included studies**

[1] AGARWAL S, PRESCIUTTI A, ROTH W, et al. Determinants of Long-Term Neurological Recovery Patterns Relative to Hospital Discharge Among Cardiac Arrest Survivors [J]. Critical care medicine, 2018, 46(2): e141-e50.

[2] ARRICH J. Clinical application of mild therapeutic hypothermia after cardiac arrest [J]. Critical care medicine, 2007, 35(4): 1041-7.

[3] BLUMENSTEIN J, LEICK J, LIEBETRAU C, et al. Extracorporeal life support in cardiovascular patients with observed refractory in-hospital cardiac arrest is associated with favourable short and long-term outcomes: A propensity-matched analysis [J]. European heart journal Acute cardiovascular care, 2016, 5(7): 13-22.

[4] CASTRéN M, NORDBERG P, SVENSSON L, et al. Intra-arrest transnasal evaporative cooling: a randomized, prehospital, multicenter study (PRINCE: Pre-ROSC IntraNasal Cooling Effectiveness) [J]. Circulation, 2010, 122(7): 729-36.

[5] CHEN Y S, LIN J W, YU H Y, et al. Cardiopulmonary resuscitation with assisted extracorporeal life-support versus conventional cardiopulmonary resuscitation in adults with in-hospital cardiac arrest: an observational study and propensity analysis [J]. Lancet (London, England), 2008, 372(9638): 554-61.

[6] CHOI D H, KIM Y J, RYOO S M, et al. Extracorporeal cardiopulmonary resuscitation among patients with out-of-hospital cardiac arrest [J]. Clinical and experimental emergency medicine, 2016, 3(3): 132-8.

[7] CHOU T H, FANG C C, YEN Z S, et al. An observational study of extracorporeal CPR for in-hospital cardiac arrest secondary to myocardial infarction [J]. Emergency medicine journal : EMJ, 2014, 31(6): 441-7.

[8] DE FAZIO C, SKRIFVARS M B, SøREIDE E, et al. Intravascular versus surface cooling for targeted temperature management after out-of-hospital cardiac arrest: an analysis of the TTH48 trial [J]. Critical care (London, England), 2019, 23(1): 61.

[9] DANKIEWICZ J, CRONBERG T, LILJA G, et al. Hypothermia versus Normothermia after Out-of-Hospital Cardiac Arrest [J]. The New England journal of medicine, 2021, 384(24): 2283-94.

[10] FERREIRA I, SCHUTTE M, OOSTERLOO E, et al. Therapeutic mild hypothermia improves outcome after out-of-hospital cardiac arrest [J]. Netherlands heart journal : monthly journal of the Netherlands Society of Cardiology and the Netherlands Heart Foundation, 2009, 17(10): 378-84.

[11] FINK K, SCHWAB T, BODE C, et al. [Endovascular or surface cooling?: therapeutic hypothermia after cardiac arrest] [J]. Der Anaesthesist, 2008, 57(12): 1155-60.

[12] FJøLNER J, GREISEN J, JøRGENSEN M R, et al. Extracorporeal cardiopulmonary resuscitation after out-of-hospital cardiac arrest in a Danish health region [J]. Acta anaesthesiologica Scandinavica, 2017, 61(2): 176-85.

[13] GILLIES M A, PRATT R, WHITELEY C, et al. Therapeutic hypothermia after cardiac arrest: a retrospective comparison of surface and endovascular cooling techniques [J]. Resuscitation, 2010, 81(9): 1117-22.

[14] GOTO T, MORITA S, KITAMURA T, et al. Impact of extracorporeal cardiopulmonary resuscitation on outcomes of elderly patients who had out-of-hospital cardiac arrests: a single-centre retrospective analysis [J]. BMJ open, 2018, 8(5): e019811.

[15] HACHIMI-IDRISSI S, ZIZI M, NGUYEN D N, et al. The evolution of serum astroglial S-100 beta protein in patients with cardiac arrest treated with mild hypothermia [J]. Resuscitation, 2005, 64(2): 187-92.

[16] HAN K S, KIM S J, LEE E J, et al. Experience of extracorporeal cardiopulmonary resuscitation in a refractory cardiac arrest patient at the emergency department [J]. Clinical cardiology, 2019, 42(4): 459-66.

[17] Mild therapeutic hypothermia to improve the neurologic outcome after cardiac arrest [J]. The New England journal of medicine, 2002, 346(8): 549-56.

[18] JOUFFROY R, LAMHAUT L, GUYARD A, et al. Early detection of brain death using the Bispectral Index (BIS) in patients treated by extracorporeal cardiopulmonary resuscitation (E-CPR) for refractory cardiac arrest [J]. Resuscitation, 2017, 120(8-13.

[19] KAGAWA E, INOUE I, KAWAGOE T, et al. Assessment of outcomes and differences between in- and out-of-hospital cardiac arrest patients treated with cardiopulmonary resuscitation using extracorporeal life support [J]. Resuscitation, 2010, 81(8): 968-73.

[20] KAGAWA E, DOTE K, KATO M, et al. Should we emergently revascularize occluded coronaries for cardiac arrest?: rapid-response extracorporeal membrane oxygenation and intra-arrest percutaneous coronary intervention [J]. Circulation, 2012, 126(13): 1605-13.

[21] KAGAWA E, DOTE K, KATO M, et al. Do Lower Target Temperatures or Prolonged Cooling Provide Improved Outcomes for Comatose Survivors of Cardiac Arrest Treated With Hypothermia? [J]. Journal of the American Heart Association, 2015, 4(9): e002123.

[22] KäMäRäINEN A, VIRKKUNEN I, TENHUNEN J, et al. Prehospital therapeutic hypothermia for comatose survivors of cardiac arrest: a randomized controlled trial [J]. Acta anaesthesiologica Scandinavica, 2009, 53(7): 900-7.

[23] KIM F, OLSUFKA M, LONGSTRETH W T, JR., et al. Pilot randomized clinical trial of prehospital induction of mild hypothermia in out-of-hospital cardiac arrest patients with a rapid infusion of 4 degrees C normal saline [J]. Circulation, 2007, 115(24): 3064-70.

[24] KIM S J, JUNG J S, PARK J H, et al. An optimal transition time to extracorporeal cardiopulmonary resuscitation for predicting good neurological outcome in patients with out-of-hospital cardiac arrest: a propensity-matched study [J]. Critical care (London, England), 2014, 18(5): 535.

[25] KIM Y S, CHO Y H, SUNG K, et al. Target Temperature Management May Not Improve Clinical Outcomes of Extracorporeal Cardiopulmonary Resuscitation [J]. Journal of intensive care medicine, 2019, 34(10): 790-6.

[26] LOOK X, LI H, NG M, et al. Randomized controlled trial of internal and external targeted temperature management methods in post- cardiac arrest patients [J]. The American journal of emergency medicine, 2018, 36(1): 66-72.

[27] MAEKAWA K, TANNO K, HASE M, et al. Extracorporeal cardiopulmonary resuscitation for patients with out-of-hospital cardiac arrest of cardiac origin: a propensity-matched study and predictor analysis [J]. Critical care medicine, 2013, 41(5): 1186-96.

[28] MECKLENBURG A, STAMM J, ANGRIMAN F, et al. Impact of therapeutic hypothermia on bleeding events in adult patients treated with extracorporeal life support peri-cardiac arrest [J]. Journal of critical care, 2021, 62(12-8.

[29] NAGAO K, KIKUSHIMA K, WATANABE K, et al. Early induction of hypothermia during cardiac arrest improves neurological outcomes in patients with out-of-hospital cardiac arrest who undergo emergency cardiopulmonary bypass and percutaneous coronary intervention [J]. Circulation journal : official journal of the Japanese Circulation Society, 2010, 74(1): 77-85.

[30] NIELSEN N, WETTERSLEV J, CRONBERG T, et al. Targeted temperature management at 33°C versus 36°C after cardiac arrest [J]. The New England journal of medicine, 2013, 369(23): 2197-206.

[31] OKADA K, OHDE S, OTANI N, et al. Prediction protocol for neurological outcome for survivors of out-of-hospital cardiac arrest treated with targeted temperature management [J]. Resuscitation, 2012, 83(6): 734-9.

[32] OTANI T, SAWANO H, NATSUKAWA T, et al. Low-flow time is associated with a favorable neurological outcome in out-of-hospital cardiac arrest patients resuscitated with extracorporeal cardiopulmonary resuscitation [J]. Journal of critical care, 2018, 48(15-20.

[33] PANG P Y, WEE G H, HOO A E, et al. Therapeutic hypothermia in adult patients receiving extracorporeal life support: early results of a randomized controlled study [J]. Journal of cardiothoracic surgery, 2016, 11(43.

[34] PANG P Y K, WEE G H L, HUANG M J, et al. Therapeutic Hypothermia May Improve Neurological Outcomes in Extracorporeal Life Support for Adult Cardiac Arrest [J]. Heart, lung & circulation, 2017, 26(8): 817-24.

[35] RYU J A, CHUNG C R, CHO Y H, et al. Neurologic Outcomes in Patients Who Undergo Extracorporeal Cardiopulmonary Resuscitation [J]. The Annals of thoracic surgery, 2019, 108(3): 749-55.

[36] SCALES D C, CHESKES S, VERBEEK P R, et al. Prehospital cooling to improve successful targeted temperature management after cardiac arrest: A randomized controlled trial [J]. Resuscitation, 2017, 121(187-94.

[37] SCHENFELD E M, STUDNEK J, HEFFNER A C, et al. Effect of prehospital initiation of therapeutic hypothermia in adults with cardiac arrest on time-to-target temperature [J]. Cjem, 2015, 17(3): 240-7.

[38] SCHOBER A, STERZ F, HERKNER H, et al. Emergency extracorporeal life support and ongoing resuscitation: a retrospective comparison for refractory out-of-hospital cardiac arrest [J]. Emergency medicine journal : EMJ, 2017, 34(5): 277-81.

[39] SHIN T G, JO I J, SIM M S, et al. Two-year survival and neurological outcome of in-hospital cardiac arrest patients rescued by extracorporeal cardiopulmonary resuscitation [J]. International journal of cardiology, 2013, 168(4): 3424-30.

[40] SONDER P, JANSSENS G N, BEISHUIZEN A, et al. Efficacy of different cooling technologies for therapeutic temperature management: A prospective intervention study [J]. Resuscitation, 2018, 124(14-20.

[41] TøMTE Ø, DRæGNI T, MANGSCHAU A, et al. A comparison of intravascular and surface cooling techniques in comatose cardiac arrest survivors [J]. Critical care medicine, 2011, 39(3): 443-9.

[42] YUKAWA T, KASHIURA M, SUGIYAMA K, et al. Neurological outcomes and duration from cardiac arrest to the initiation of extracorporeal membrane oxygenation in patients with out-of-hospital cardiac arrest: a retrospective study [J]. Scandinavian journal of trauma, resuscitation and emergency medicine, 2017, 25(1): 95.
